# Single‐Cell Resolution Mapping of Osteoarthritis‐Associated Chondrocyte Dynamics Using a High‐Density CMOS MEA Platform

**DOI:** 10.1002/adhm.202504115

**Published:** 2026-10-14

**Authors:** Neha Deshpande, Pauline Coquart, Bram Theuns, Susanne Cremery, Jaime Rodolfo V. Garcia III, Julien Verplanken, Dennis Lambrechts, Dries Braeken, Liesbet Lagae, Johanna Bolander

**Affiliations:** ^1^ imec Leuven Belgium; ^2^ Department of Physics and Astronomy KU Leuven Leuven Belgium; ^3^ BIH Center for Regenerative Therapies Berlin Institute of Health at Charité ‐ Universitätsmedizin Berlin Germany; ^4^ Julius Wolff Institute Berlin Institute of Health at Charité ‐ Universitätsmedizin Berlin Germany

**Keywords:** chondrocytes, CMOS MEA, impedance, osteoarthritis, single‐cell biosensing

## Abstract

Fibrosis is a major cause of chronic tissue dysfunction and contributes substantially to morbidity and mortality worldwide. Osteoarthritis (OA), affecting over 590 million people, is a prevalent fibrotic joint disease for which disease‐modifying therapies remain lacking, partly because disease progression remains incompletely understood. As human‐relevant in vitro models, including organ‐on‐chip and microphysiological systems, become increasingly important, non‐invasive technologies capable of continuous cellular monitoring are urgently needed. Here, we implement high‐density complementary metal‐oxide‐semiconductor (CMOS) multielectrode array (MEA)‐based impedance profiling as a label‐free platform for longitudinal assessment of OA‐relevant chondrocyte remodeling. Human primary chondrocytes cultured on CMOS‐MEA chips exhibited impedance increases that correlated with electrode occupancy and extracellular matrix (ECM) deposition. Spatial impedance mapping enabled visualization of injury‐response dynamics, while oxidative stress induced progressive impedance decline accompanied by apoptosis, morphological remodeling, and chondrocyte dedifferentiation, evidenced by reduced collagen II and SOX9 expression and increased collagen I expression. In contrast, osteogenic de‐differentiation produced increased impedance associated with ECM remodeling and matrix mineralization. Together, these findings support high‐density CMOS‐MEA impedance profiling as a non‐invasive approach for monitoring cellular remodeling and provide a foundation for future integration of spatially resolved impedance sensing into advanced microphysiological systems for OA and other fibrotic diseases.

## Graphical abstract

Single‐cell and spatially resolved impedance mapping using a high‐density CMOS‐MEA platform enables non‐invasive monitoring of osteoarthritis‐associated chondrocyte dynamics. Continuous label‐free measurements reveal heterogeneous remodeling behaviors, including proliferation, migration, and phenotypic transitions, establishing a powerful approach for studying cartilage‐associated pathobiology and assessing therapeutic responses.

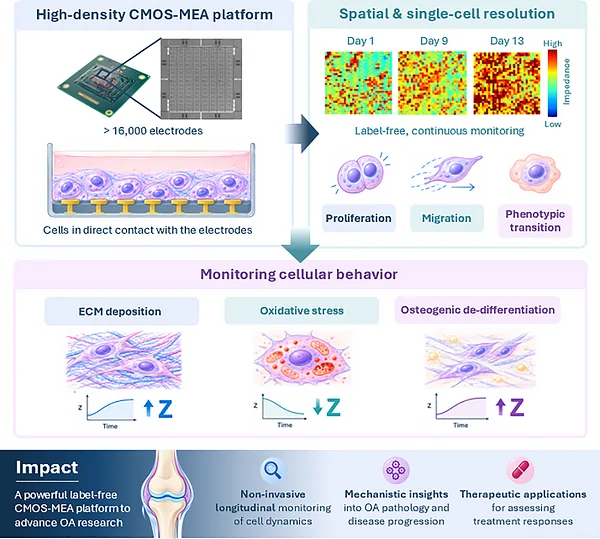

## 1 Introduction

Through uncontrolled extracellular matrix (ECM) deposition, fibrosis undermines the structural and functional integrity of tissues and remains a major contributor to morbidity and mortality across diverse organ systems [1]. This pathological process is central to many chronic inflammatory diseases, including osteoarthritis (OA). In OA, fibrotic remodeling alters cellular behavior and impairs joint function, leading to joint stiffness and pain [2, 3]. The human synovial knee joint comprises multiple specialized tissues, including the articular cartilage covering the tibial and femoral surfaces, the synovial membrane, the Hoffa fat pad, and the subchondral bone [2, 4]. This complex structure is biomechanically optimized to facilitate frictionless and pain‐free motion throughout life. However, the avascular nature of articular cartilage limits its regenerative capacity, rendering it particularly vulnerable to physical or mechanical trauma. Injuries to the cartilage typically fail to heal, instead initiating fibrotic cascades and scar tissue formation [4]. Indeed, several pathophysiological processes—including inflammation, tissue degeneration, maladaptive repair mechanisms, and metabolic dysregulation—interact and reinforce one another, driving fibrotic progression and underscoring the multifactorial nature of OA [5].

Articular cartilage is composed of chondrocytes embedded within a highly organized ECM rich in collagen type II (COL2) and proteoglycans, components that confer the tissue's unique viscoelastic and load‐bearing properties [6, 7]. During OA progression, chondrocytes undergo profound phenotypic alterations that disrupt the balance between matrix synthesis and degradation [8, 9, 10]. Exposure to OA‐associated stressors, including excessive mechanical loading, inflammatory cytokines, and oxidative stress, promotes cellular dysfunction, senescence, and apoptosis [8, 9, 10]. Chondrocytes further acquire a fibroblast‐like phenotype characterized by reduced COL2 expression and increased collagen type I (COL1) production, contributing to fibrocartilage formation and loss of tissue functionality [10, 11, 12]. Oxidative stress, driven by excessive accumulation of reactive oxygen species (ROS), is a particularly important mediator of OA pathology, impairing proliferation, migration, and regenerative capacity while amplifying inflammatory signaling [13, 14, 15]. In addition, growing evidence suggests that OA chondrocytes may undergo hypertrophic and osteogenic reprogramming reminiscent of endochondral ossification, contributing to cartilage calcification and disease progression [16, 17, 18]. Thus, OA progression is primarily driven by persistent, unresolved stress response triggered by OA‐associated insults and inflammation, contributing to phenotypic transitions within chondrocytes.

Nevertheless, the molecular mechanisms governing OA onset and progression remain incompletely understood, contributing to the continued absence of disease‐modifying therapies [19]. To accelerate research progress and reduce reliance on animal experimentation, regulatory agencies and funding bodies are increasingly advocating the adoption of human‐relevant in vitro platforms, including microphysiological systems (MPSs) and organ‐on‐chip (OoC) technologies, for fundamental research, drug development, and efficacy testing [20, 21]. However, an often‐overlooked limitation across both conventional in vitro models and emerging MPS platforms is their continued reliance on destructive endpoint assays. Consequently, cellular responses are typically assessed through static snapshots obtained from independent samples at discrete timepoints, precluding continuous longitudinal assessment of the same biological system [22]. The absence of integrated non‐invasive monitoring technologies limits our ability to investigate the temporal evolution of disease‐associated phenotypes, characterize dynamic cellular responses to stress and therapeutic interventions, and identify early cellular changes that precede later pathological outcomes. Addressing this technological gap would substantially enhance the utility of both simple reductionist models and increasingly complex, human in vitro systems for mechanistic studies and therapeutic discovery.

Recently, considerable effort has been devoted to integrating real‐time sensing modalities within MPS platforms, including optical, mechanical, biochemical, and electrical approaches [23, 24, 25], also in joint‐on‐chips [26]. Optical sensing exploits the transparency of microfluidic materials for non‐invasive monitoring of cellular activity and tissue architecture, while mechanical sensing enables the quantification of strain, stress, and contractile forces. However, both approaches face challenges related to image processing, limited tissue penetration, or sensor integration complexity. Biochemical sensors further enable the continuous detection of metabolites and secreted biomarkers, but technology is currently limited by biofouling and long‐term stability. Among these technologies, electrical sensing techniques, including electrical impedance spectroscopy (EIS), electric cell‐substrate impedance sensing (ECIS), trans endothelial/epithelial electrical resistance (TEER), and multielectrode arrays (MEAs) provide label‐free, high‐temporal‐resolution monitoring of barrier integrity, cell–substrate interactions, and electrophysiological activity, making them particularly attractive for continuous functional assessment of MPS platforms. Impedance‐based sensing offers a promising strategy for non‐invasive, label‐free monitoring of dynamic cellular processes, providing a continuous functional readout of cellular state without perturbing the culture [27, 28]. Historically, conventional impedance sensing has relied on low‐density, planar electrode configurations, relying on one or a few relatively large electrodes to produce a single spatially averaged impedance signal that represented the collective behavior of the entire cell population [29, 30, 31]. Advances in CMOS‐MEA technology now enable semiconductor‐scale monolithic integration of thousands to tens of thousands of individually addressable microelectrodes with parallel readout electronics, impedance spectroscopy, electrical stimulation, and electrophysiological recording on a single chip [27, 32, 33]. These highly integrated platforms provide unprecedented high‐throughput, temporally and spatially resolved impedance imaging with multimodal and scalable integration, making them ideally suited for continuous, non‐invasive monitoring of not only conventional in vitro models but also complex MPS and OoC models. We have previously developed a state‐of‐the‐art CMOS MEA platform featuring high‐density electrode arrays capable of monitoring single‐cell neuronal action potentials [32, 33, 34]. Compared to conventional commercial systems, this advanced platform offers significantly reduced noise levels, supports multimodal measurements, and enhances overall experimental throughput and flexibility [32, 33, 34]. The CMOS MEA chip comprises 16,384 titanium nitride (TiN) electrodes, distributed across 16 active regions. Each region consists of a 32 × 32 electrode array (1,024 electrodes) with a 15 µm pitch and 8 µm electrode diameter. The system integrates 1,024 parallel readout channels, 64 multiplexers, 64 stimulation units, and 64 analog‐to‐digital converters (ADCs), enabling simultaneous acquisition of diverse electrical signals. In addition to electrophysiological recordings and electrical stimulation, the chip is equipped for fixed‐frequency impedance monitoring and electrical impedance spectroscopy, providing a comprehensive suite of capabilities for monitoring dynamic cellular responses even in non‐electrogenic cells [32, 33, 34]. Furthermore, the design and fabrication processes have been extensively optimized to address common challenges with MEA chips, such as spatial constraints, thermal dissipation, sensor‐to‐signal‐source proximity, and cytotoxicity, ensuring compatibility with live‐cell applications.

This study evaluates high‐density CMOS‐MEA impedance profiling as a non‐invasive approach to monitor OA‐relevant chondrocyte remodeling in vitro. Using complementary optical, viability, ECM, and immunocytochemical assays, we assessed how spatial impedance patterns relate to cell coverage, adhesion, migration, oxidative‐stress responses, ECM remodeling, and phenotypic marker changes. Thus, impedance signal serves as a composite, context‐dependent readout of critical biological processes. These data establish a foundation for applying spatially resolved impedance mapping to dynamic cell cultures and, in future work, to more complex human MPSs with the goal of studying human degenerative diseases.

## 2 Results

### 2.1 Label‐Free Impedance Monitoring Uncovers Chondrocyte Proliferation and Growth Dynamics

To study the phenotype and cell characteristics of chondrocytes during in vitro modeling of OA non‐invasively and with spatial resolution, we investigated the utility of CMOS MEA‐based impedance monitoring to quantitatively assess the cellular characteristics. For this, human chondrocytes were cultured on poly‐L‐lysine (PLL)‐coated CMOS MEA chips (Figure S1A). The base impedance of uncoated chips is 0.211 ± 0.04 MΩ and that of PLL‐coated chips is 0.210 ± 0.04 MΩ, suggesting that coating of the electrode surfaces with PLL did not alter their base impedance (Figure S1B). Based on a frequency sweep, 1 kHz and 1 nA were selected as optimal parameters to target the impedance range most sensitive to cell monolayer integrity for all further experiments (Figure S1E). Owing to the high‐density placement of electrodes on the active culture area of the chip, proportional increases in impedance were expected in response to cellular coverage of the electrodes. This was investigated by seeding chondrocytes at different seeding densities on CMOS‐MEA chips. In addition to metabolic activity as a surrogate for cell number, nuclear counts and electrode coverage were quantified from fluorescence images and compared directly with spatial impedance maps. Quantification of fluorescent images demonstrated a progressive increase in nuclei count and cell coverage with increasing seeding density, while PrestoBlue measurements showed a corresponding increase in metabolic activity (Figures 1A,B, S1C, and S1D). Fixed‐frequency impedance measurements at 1 kHz and 1 nA demonstrated a density‐dependent increase in impedance (5k/chips: Z = 0.354 ± 0.202 MΩ; 10k/chips: Z = 0.494 ± 0.259 MΩ; 15k/chips: Z = 0.576 ± 0.287 MΩ; 20k/chips: Z = 0.634 ± 0.282 MΩ) (Figure 1C). Comparison of the impedance heatmaps with fluorescence microscopy demonstrated that regions of elevated impedance corresponded to increased electrode occupancy by cells at higher seeding densities (Figure 1A–C), supporting a positive relationship between impedance and cellular occupancy.

**FIGURE 1:**
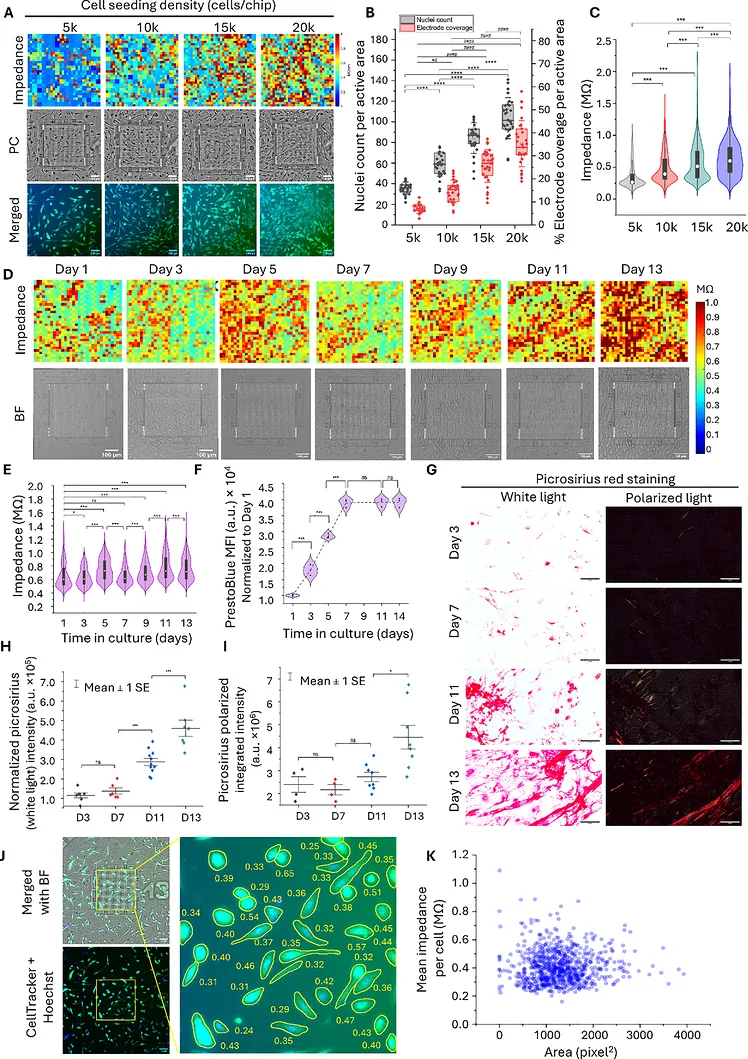
Proliferation and growth dynamics of chondrocytes on CMOS MEA chips. Chondrocytes labeled with Hoechst 33342 and CellTracker Green were seeded onto CMOS MEA chips at seeding densities of 5, 10, 15, and 20 × 10^3^ cells per chip. (A) Impedance heatmaps alongside corresponding BF, PC, and fluorescence images illustrate the spatial distribution of cells at each density. (B) Quantification of nuclear counts and electrode coverage per active area confirmed progressively greater cell occupancy with increasing seeding density. (C) Violin plots with nestled box plots demonstrate density‐dependent increases in impedance values. (D) Time‐course spatial impedance heatmaps and corresponding BF images show progressive changes in cell coverage across days. Scale bars denote 100 µm. (E) Violin plots with nestled box plots quantify temporal changes in impedance values. Statistics for comparisons between consecutive time points and between day 1 and subsequent time points are displayed in the graph. Comparisons between all other time points are available in Supporting Table S1. (F) Cell viability, assessed in parallel using PrestoBlue assay, demonstrates exponential chondrocyte proliferation till day 7, when confluency was reached. Absence of further increases in PrestoBlue fluorescence indicates contact inhibition. (G) White light microscopy and polarized light microscopy‐based representative images of picrosirius red staining revealed increased collagen fibrils at day 11 and day 13 of chondrocyte culture. Scale bar denotes 100 µm; *n* = 2. (H–I) Scatter plots reveal quantification of (H) picrosirius staining intensity from light microscopy‐based images normalized to the number of cells and (I) integrated intensity from polarized microscopy‐based images; *n* = 2; *N* = 300 cells. (J) Representative fluorescent image zoomed in on the active area map of an individual cell and depicts mean impedance per cell (values in yellow; MΩ). (K) Scatter plot reveals no correlation between cell area and mean impedance per cell; *n* = 3; *N* = 817 cells. All experiments were conducted in triplicates, with >800 cells and >1000 electrodes, unless otherwise stated. *P*‐values < 0.05, 0.01, 0.001, and 0.0001 are represented by *, **, ***, and **** or by #, ##, ###, and ####, respectively. A.U., arbitrary units; BF, brightfield; CI, confidence interval; MFI, mean fluorescence intensity; PC, phase contrast; and SE, standard error.

To dynamically assess chondrocyte growth and culture development over time, cells seeded on chips were subjected to fixed‐frequency impedance measurements (1 kHz, 1 nA) every 2 days for a period of 13 days. A time‐dependent increase in impedance was observed until day 5 with a transient decrease observed on day 7 (Day 1: Z = 0.655 ± 0.189 MΩ; day 3: 0.634 ± 0.137 MΩ; day 5: 0.749 ± 0.164 MΩ; day 7: 0.650 ± 0.141 MΩ; day 9: 0.703 ± 0.157 MΩ; day 11: 0.784 ± 0.203 MΩ; and day 13: 0.755 ± 0.182 MΩ) (Figure 1D,E). The increase in impedance coincided with progressive expansion of cellular coverage across the electrode surface, as observed by brightfield microscopy (Figure 1D–F), consistent with increasing resistance to current flow through the cell‐covered regions. After cultures reached confluency around day 7, further increases in metabolic activity plateaued in the PrestoBlue assay (Figure 1F), consistent with contact inhibition limiting further proliferation. However, progressive increases in impedance were observed until day 13 (Figure 1D,E), potentially reflecting increased anabolic and secretory activity of chondrocytes, particularly ECM synthesis and deposition. Picrosirius red assay was undertaken to directly assess whether further increases in impedance corresponded to increased collagen synthesis. Light and polarized microscopy demonstrated progressive collagen fibril deposition throughout the culture period (Figure 1G–I), supporting the interpretation that continued ECM accumulation contributed to the sustained impedance increase after confluency. Collectively, these findings indicate that the temporal impedance profile reflects multiple aspects of chondrocyte culture development, including expansion of cellular coverage during early growth and continued ECM deposition following confluency. The observed impedance progression closely paralleled the characteristic in vitro growth kinetics of chondrocytes, demonstrating the utility of CMOS‐MEA impedance monitoring for longitudinal assessment of chondrocyte dynamics.

Our CMOS high‐density MEA chips contain 16 active areas, each comprising 1,048 electrodes evenly distributed across a 480 × 480 µm^2^ area, enabling single‐cell impedance measurements. To evaluate whether impedance measurements could be resolved at the level of individual cells, we next developed a multimodular image‐analysis workflow that mapped fluorescently identified chondrocytes to their underlying electrodes. This mapping enabled the calculation of mean impedance per cell (Figure 1J). The distribution of impedance values per chondrocyte followed a Gamma distribution (shape, a = 5.405; scale, *θ* = 0.048; loc = 0.15) with mild positive skew (Figure S1F). No significant correlation was observed between projected two‐dimensional cell area and mean cellular impedance (Figure 1K), indicating that cell size alone does not determine the measured impedance signal. These results demonstrate the feasibility of assigning impedance measurements to individual cells while preserving their spatial context within the culture, establishing a foundation for future investigations of spatially resolved cellular heterogeneity and dynamic phenotypic responses.

### 2.2 Spatiotemporal Impedance Mapping Enables Real‐Time Monitoring of Wound Closure and Dynamic Cell–Substrate Interactions

Cell migration is a crucial cellular response during tissue regeneration following tissue trauma [35]. Therefore, we next explored the utility of CMOS MEA‐based impedance recording as a spatial and real‐time electrical monitoring platform to track migration‐associated cellular redistribution. For this, a migration assay, where a scratch wound is created in a confluent cell monolayer, was performed, and the movement of cells into the cleared area was monitored over time to assess migratory capacity. The assay was performed on a confluent layer of chondrocytes cultured on CMOS MEA chips. Heatmaps derived from impedance measurements revealed a gradual recovery of impedance within the scratched region over time, consistent with progressive repopulation of the wound area (Figure 2A). In active areas that were half‐ or completely scratched, a sharp decline in impedance was observed immediately at 0 h, which gradually increased after 18 h, with significant recovery at 48 h (t = −1 h, Z = = 0.715 ± 0.150 MΩ; t = 0 h, Z = 0.602 ± 0.108 MΩ; t = 6 h, Z = 0.552 ± 0.09 MΩ; t = 12 h, Z = 0.538 ± 0.087 MΩ; t = 18 h, Z = 0.508 ± 0.103 MΩ; t = 24 h, Z = 0.576 ±  0.096 MΩ; and t = 48 h, Z = 0.647 ± 0.133 MΩ) (Figure 2B). A similar trend was observed in the full‐scratched active area (t = −1 h, Z = 0.73 ± 0.154 MΩ; t = 0 h, Z = 0.561 ± 0.048 MΩ; t = 6 h, Z = 0.504 ± 0.059 MΩ; t = 12 h, Z = 0.488 ± 0.043 MΩ; t = 18 h, Z = 0.480 ± 0.068 MΩ; t = 24 h, Z = 0.506 ± 0.070 MΩ; and t = 48, Z = 0.619 ± 0.147 MΩ) (Figure 2C). Concordantly, representative brightfield images from parallel wound‐healing assays demonstrated progressive wound closure over the same time period (Figure 2E), consistent with the temporal evolution observed in the impedance maps. Interestingly, in the confluent active area adjacent to the scratch, impedance remain unaffected at 0 h immediately post‐injury; however, a gradual decrease in impedance was observed as early as 6 h, which recovered completely at 48 h (t = −1 h, Z = 0.784 ± 0.16 MΩ; t = 0 h, Z = 0.792 ± 0.158 MΩ; t = 6 h, Z = 0.717 ± 0.107 MΩ; t = 12 h, Z = 0.693 ± 0.091 MΩ; t = 18 h, Z = 0.698 ± 0.082 MΩ; t = 24 h, Z = 0.736 ± 0.077 MΩ; and t = 48, Z = 0.845 ± 0.120 MΩ) (Figure 2D). The transient impedance decrease observed in the adjacent confluent region likely reflects dynamic redistribution of cells toward the wound area, accompanied by changes in local cell–electrode interactions during wound closure.

**FIGURE 2:**
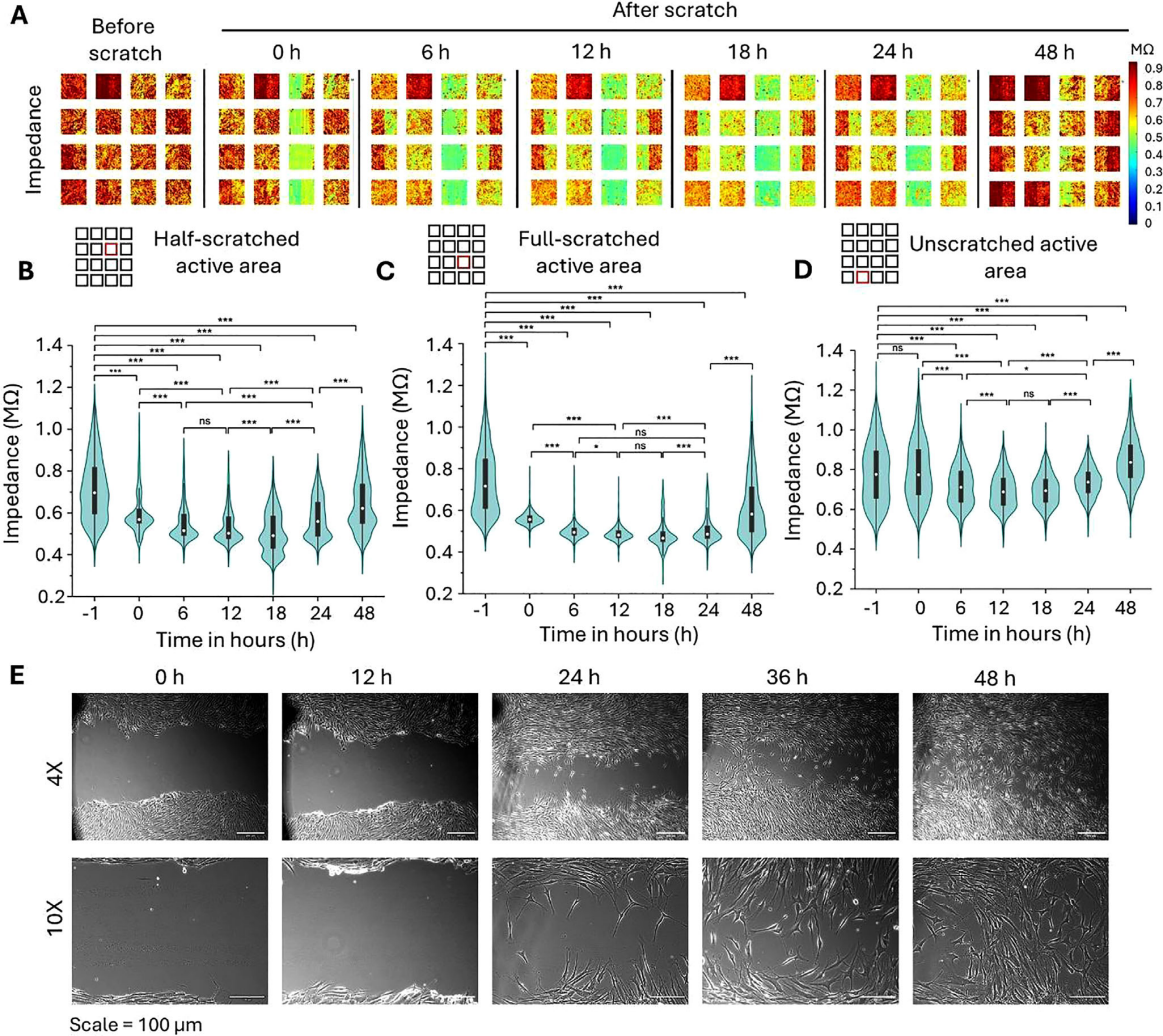
Spatial and real‐time impedance monitoring tracks chondrocyte migration. A scratch wound was created on a confluent layer of chondrocytes cultured on active CMOS MEA chips. (A) Impedance heatmaps depict cell migration kinetics at specified time points post wound creation. Violin plots with nested box plots reflect temporal impedance changes from specified active areas of the CMOS MEA chips that were either (B) half‐scratched, (C) completely scratched, or (D) unscratched adjacent to the wound area. (E) Phase contrast microscopy of parallel scratch assays conducted on Ibidi inserts captured chondrocyte migration at specified time points at 4X and 10X magnification. Scale bars denote 100 µm. All experiments were conducted in triplicates, with >1000 electrodes analyzed. *P*‐values < 0.05, 0.01, and 0.001 are represented by *, **, and ***, respectively.

To validate whether CMOS MEA‐based impedance monitoring can track dynamic changes to cell adhesion, chondrocytes cultured on chips were treated with 5 mM EDTA for a period of 2.5 h. Cellular adhesion is governed by proteins that require calcium ions. Chelation of calcium ions by EDTA disrupts cell–cell and cell–substrate adhesion, resulting in rounding up of cells (Figure S2A). Indeed, EDTA treatment led to an immediate drastic decrease in impedance, with gradual decreases over time (Before, Z = 0.401 ± 0.093 MΩ; time after EDTA addition: t = 0h00 m, Z = 0.23 ± 0.059 MΩ; t = 0h15 m, Z = 0.236 ± 0.055 MΩ; t = 0h30 m, Z = 0.228 ± 0.053 MΩ; t = 0h45 m, Z = 0.220 ± 0.051 MΩ; t = 1h00 m, Z = 0.215 ± 0.051 MΩ; t = 1h15 m, Z = 0.212 ± 0.050 MΩ; t = 1h30 m, Z = 0.209 ± 0.049 MΩ; t = 1h45 m, Z = 0.209 ± 0.050 MΩ; t = 2h00 m, Z = 0.208 ± 0.04 MΩ; t = 2h15 m, Z = 0.206 ± 0.048 MΩ; t = 2h30 m, Z = 0.206 ± 0.048 MΩ) (Figure S2B,C), consistent with reduced cell–substrate interactions following EDTA‐mediated disruption of cellular adhesion. Interestingly, subsequent cell recovery following EDTA removal resulted in a progressive impedance increase, reflecting the reattachment kinetics of cells ([after EDTA removal] t = 3h00 m, Z = 0.306 ± 0.129 MΩ; t = 3h15 m, Z = 0.323 ± 0.140 MΩ; t = 3h30 m, Z = 0.333 ± 0.145 MΩ; t = 3h45 m, Z = 0.339 ± 0.148 MΩ; t = 4h00 m, Z = 0.346 ± 0.150 MΩ; t = 4h15 m, Z = 0.349 ± 0.151 MΩ; t = 5h00 m, Z = 0.367 ± 0.152 MΩ; t = 6h15 m, Z = 0.372 ± 0.152 MΩ; t = 6h30 m, Z = 0.372 ± 0.151 MΩ; t = 6h45 m, Z = 0.372 ± 0.150 MΩ; t = 8h00 m, Z = 0.415 ± 0.149 MΩ) (Figure S2B,C). Together with the scratch assay, these results demonstrate that CMOS‐MEA impedance monitoring provides a sensitive, label‐free approach for tracking dynamic changes in cell distribution and cell–substrate interactions relevant to cellular remodeling, offering a robust tool for investigating OA pathophysiology.

### 2.3 Spatiotemporal Impedance Mapping Reveals Oxidative Stress–Induced Cellular Remodeling

Oxidative stress and ECM degradation products are major cellular stressors in OA that disrupt chondrocyte homeostasis and promote phenotypic remodeling [13]. To establish whether impedance monitoring could sensitively detect stress‐induced cellular responses, 5% DMSO was first used as a positive control to induce acute cellular stress. Exposure to 5% DMSO led to chondrocyte detachment and changes in their spatial location (Figure S3A). These changes corresponded to a gradual, time‑dependent decline in cellular impedance (Before, Z = 0.450 ± 0.127 MΩ; t = 0h00 m, Z = 0.397 ± 0.120 MΩ; t = 0h15 m, Z = 0.359 ± 0.105 MΩ; t = 0h30 m, Z = 0.358 ± 0.104 MΩ; t = 0h45 m, Z = 0.340 ± 0.095 MΩ; t = 1h00 m, Z = 0.327 ± 0.090 MΩ; t = 1h15 m, Z = 0.329 ± 0.087 MΩ; t = 1h30 m, Z = 0.319 ± 0.081 MΩ; t = 1h45 m, Z = 0.314 ± 0.076 MΩ; t = 2h00 m, Z = 0.311 ± 0.074 MΩ; t = 2h30 m, Z = 0.302 ± 0.068 MΩ; t = 2h45 m, Z = 0.303 ± 0.066 MΩ; t = 3h00 m, Z = 0.297 ± 0.063 MΩ; t = 3h15 m, Z = 0.291 ± 0.061 MΩ; t = 3h30 m, Z = 0.286 ± 0.061 MΩ; t = 3h45 m, Z = 0.275 ± 0.059 MΩ; t = 4h00 m, Z = 0.273 ± 0.059 MΩ) (Figures S3B–C). The temporal profile of impedance reduction, visualized in the spatial impedance heatmap (Figure S3B), was consistent with the progressive redistribution of cells toward the bottom‑left quadrant of the active CMOS‑MEA area (Figure S3A and Supporting Video S1). Parallel live–dead assays revealed that the impedance decrease coincided with increased cell detachment and apoptosis (Figure S3D). Collectively, these results demonstrate that impedance monitoring provides a sensitive, non‐invasive readout of stress‐induced cellular remodeling, with impedance changes corresponding to independently validated alterations in cell viability.

Overproduction of ROS is a key feature of OA initiation, as ROS‐mediated oxidative stress triggers cellular damage and inflammation in chondrocytes, ultimately inducing fibrotic phenotypic transitions [13, 36]. Chondrocytes exhibited gradual morphological changes in response to varying concentrations of H_2_O_2_ (Figure S4A). Within the mechanically vulnerable knee synovial joint, traumatic injuries such as anterior cruciate ligament (ACL) rupture, osteochondral injuries, or meniscal damage lead to the initiation of post‐traumatic OA (PTOA) [37]. These trauma‐intense injuries, such as during ACL reconstruction are characterized by prolonged presence of activated neutrophils producing elevated acute levels of ROS [38]. Such acute oxidative stress response was simulated in vitro by exposing the cells to 1.5 mM H_2_O_2_. A significant decrease in cell area and aspect ratio was noted when chondrocytes were treated with 1.5 mM H_2_O_2_ for 1 and 4 h (Figure S4B,C). Exposure to ROS led to a time‐dependent decrease in impedance (t = before, Z = 0.426 ± 0.075 MΩ; t = 0 h, Z = 0.374 ± 0.063 MΩ; t = 1 h, Z = 0.362 ± 0.057 = MΩ; t = 2 h, Z = 0.332 ± 0.048 MΩ; t = 3 h, Z = 0.310 ± 0.044 MΩ; and t = 4 h, Z = 0.302 ± 0.042 MΩ) (Figure 3A,B). These changes correlated with observed morphological changes, including cell clustering and rounding (Figure 3C). Parallel live–dead assays confirmed that oxidative stress significantly reduced chondrocyte viability to 55.6% and 21.1% by 1 and 4 h, respectively, compared to untreated chondrocytes (Figure 3D,E). A rapid decrease in impedance characterized the early response to ROS exposure and coincided with pronounced cellular remodeling, including clustering, rounding, and reduced projected cell area (Figure 3B–D). At later time points, impedance reached a plateau that coincided with extensive cell death (Figure 3B–D). Collectively, these findings indicate that impedance acts as a composite readout of chondrocyte phenotypic state, reflecting the integrated effects of dynamic morphological remodeling, cell–substrate interactions, and cell viability rather than cell area alone. Furthermore, EIS‐based frequency sweep revealed that, at a low ROS concentration (150 µM), which simulates OA‐associated chronic ROS exposure, a decrease in impedance was observed at 1 kHz and higher frequencies in response to 24 h of ROS stimulation (Figure S4D). At 1 kHz, the impedance of chondrocytes before and after treatment with 150 µM H_2_O_2_ (ROS) for 24 h was 6.451 ± 1.374 and 5.922 ± 1.831 mΩ, respectively, whereas the base impedance of empty chips was 3.062 ± 0.177 mΩ. Altogether, our data revealed that both acute and chronic ROS exposure led to impedance reductions. Taken together, these findings demonstrate that both fixed‐frequency impedance monitoring and EIS sensitively detect ROS‐induced alterations in chondrocyte state across a range of oxidative stress conditions, supporting their utility as non‐invasive tools for longitudinal monitoring of OA‐relevant cellular remodeling.

**FIGURE 3:**
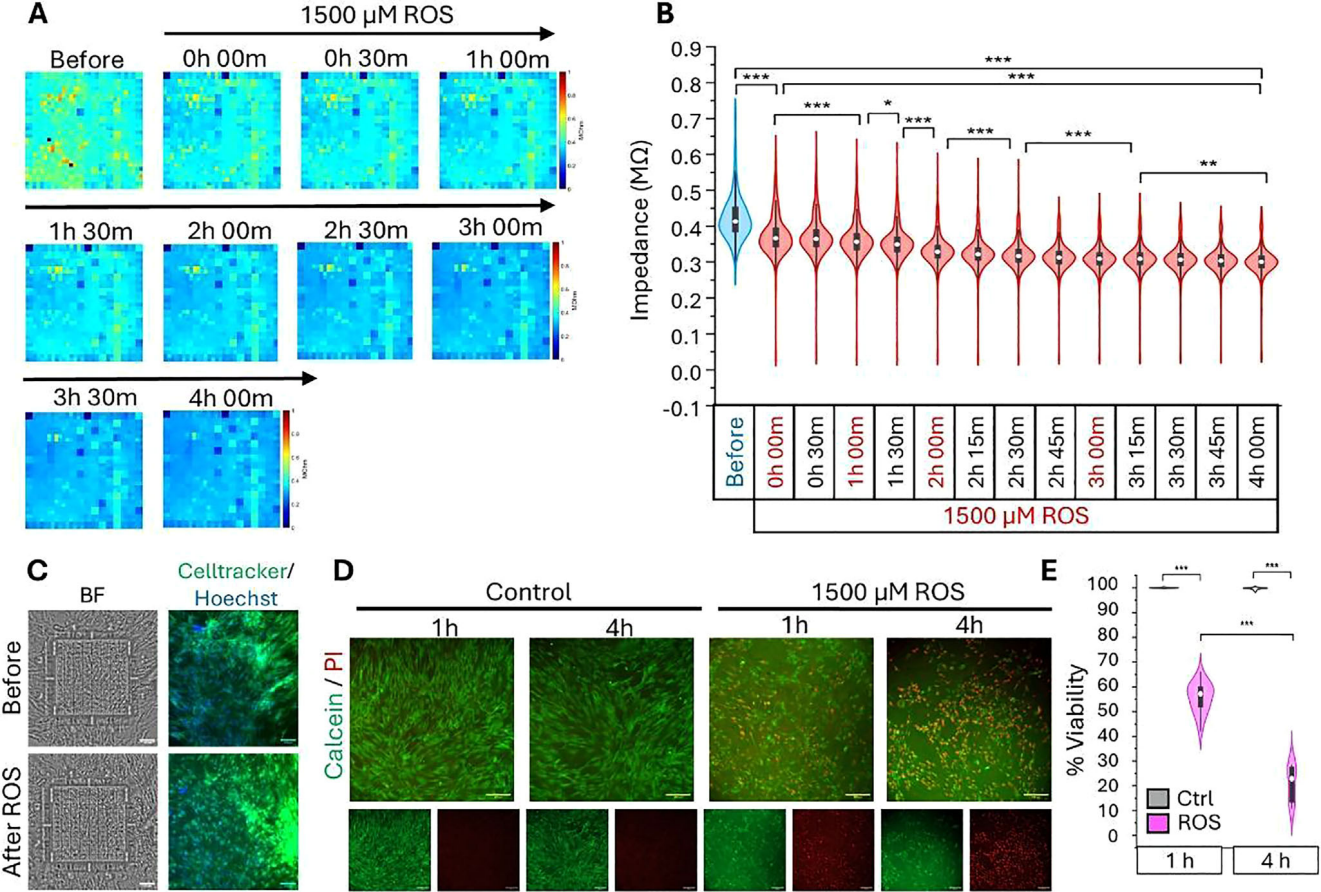
Oxidative stress induces progressive bioelectrical decline in chondrocytes. Chondrocytes seeded onto CMOS MEA chips were treated with 1.5 mM ROS for 4 h. (A) Heatmaps and (B) violin plots with nested box plots depict time‐dependent impedance decrease following ROS treatment; *n* = 3; *N* = 1024. (C) Brightfield (BF) and fluorescence images illustrate the spatial distribution and clustering of cells in response to ROS. Scale bars denote 100 µm. (D, E) Parallel live–dead assay and its quantification reveal significant ROS‐mediated cell death; *n* = 3, *N* = 15–23 FOV. *P*‐values < 0.05, 0.01, and 0.001 are represented by *, **, and ***, respectively. FOV, microscopic images/fields‐of‐view.

### 2.4 Oxidative Stress‐Induced Molecular Remodeling Corresponds With Longitudinal Impedance Changes

Under homeostatic conditions, chondrocytes residing within the articular cartilage express SRY‐box transcription factor 9 (SOX9), the key transcription factor regulating chondrogenic differentiation [39]. SOX9 promotes cartilaginous ECM synthesis rich in COL2 [40], while acting as a negative regulator of dedifferentiation and osteoblastic reprogramming by inhibiting the expression of Runt‐related transcription factor 2 (RUNX2) and COL1 [41]. Indeed, chondrocytes derived from OA patients are known to express reduced levels of SOX9 [42, 43], reflecting progressive loss of the chondrogenic phenotype. Having established that oxidative stress induces characteristic temporal impedance changes, we next investigated whether these electrical signatures were associated with established molecular markers of OA‐related chondrocyte phenotypic remodeling. Thus, to identify molecular correlates of the impedance signature, the effect of ROS on the expression of COL1, COL2, and SOX9 was assessed using immunocytochemistry. Control chondrocytes cultured in the absence of ROS‐stimulation showed robust expression of COL2—the native ECM associated within healthy chondrocytes (Figure 4A). In contrast, ROS treatment led to a decrease in the COL2 expression while concomitantly increasing COL1 expression, consistent with chondrocyte phenotypic remodeling toward a fibrocartilage‐like state (Figure 4A,B).

**FIGURE 4:**
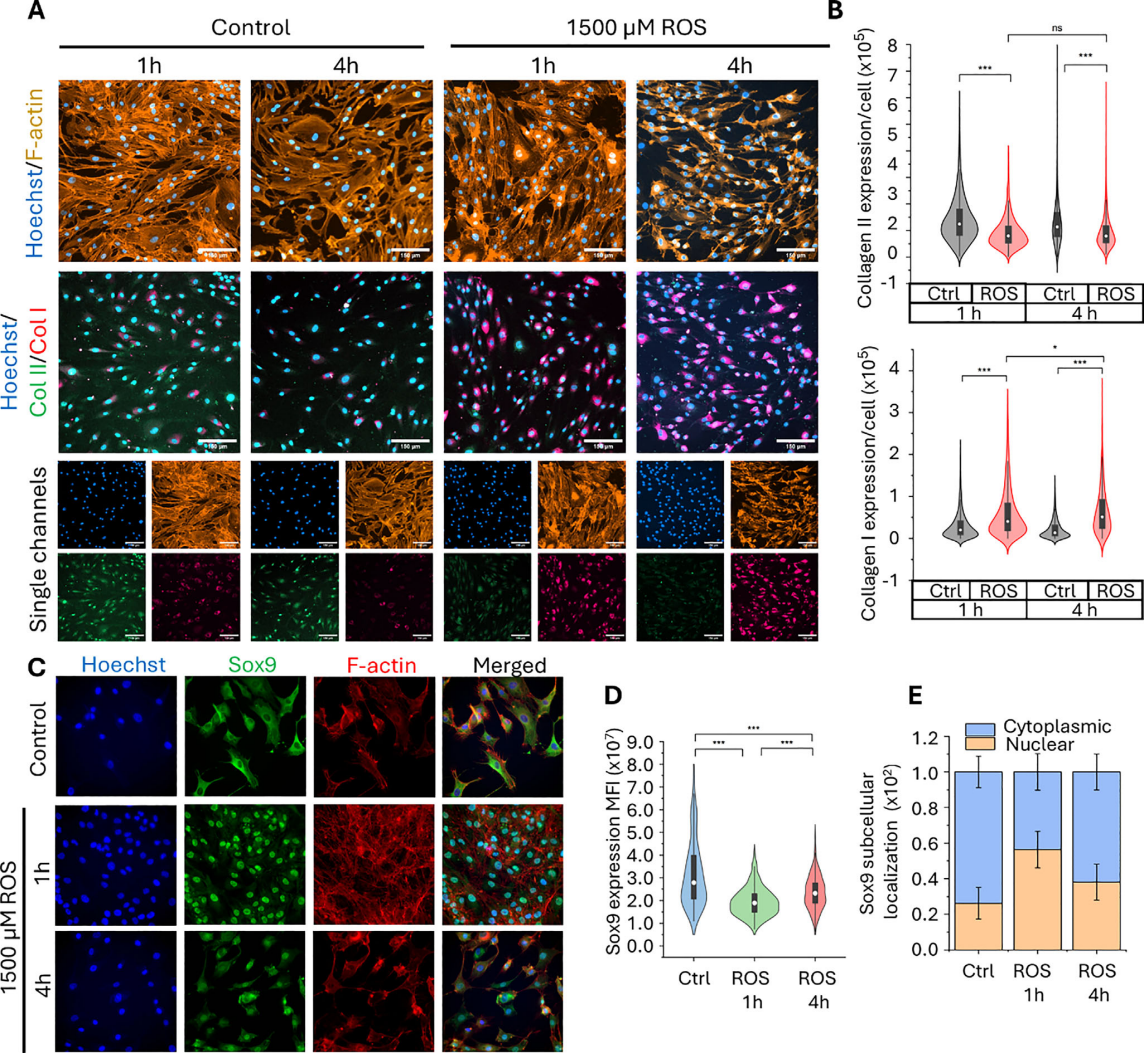
Oxidative stress disrupts chondrocyte identity via SOX9 nuclear translocation, COL2 suppression, and COL1 induction. Articular chondrocytes were either cultured untreated or treated with 1.5 mM H_2_O_2_ for 1 and 4 h. (A) Representative fluorescent images show immunostainings for COL2 and COL1. Hoechst 33342 was used to counterstain nuclei, whereas phalloidin was used for F‐actin staining. Pseudo‐colors used for COL2, COL1, F‐actin, and Hoechst are green, pink, amber, and blue, respectively. Scale bar denotes 150 µm. (B) Violin plots with nested box plots depict reduced COL2 and increased COL1 staining intensity per cell in ROS‐treated cells compared to control healthy chondrocytes; *n* = 3; *N* = >1500 cells. (C) Representative fluorescent images show SOX9 expression patterns in response to ROS treatment. Pseudo‐colors used for SOX9, F‐actin, and Hoechst are green, red, and blue, respectively. (D) Violin plot with nested box plots depicts decreased SOX9 staining intensity in ROS‐treated cells compared to control; *n* = 3; *N* = 112–134 cells. (E) Stacked bar chart depicts the nuclear and cytoplasmic localization of SOX9 per cell. Enhanced nuclear localization of SOX9 was observed at 1 h post‐ROS treatment, with gradual redistribution by 4 h; *n* = 3; *N* = 112–134 cells. *P*‐values < 0.05, 0.01, and 0.001 are represented by *, **, and ***, respectively.

In parallel, SOX9 expression was abundant in healthy control chondrocytes (Figure 4C,D) but was progressively reduced following 1 and 4 h of ROS exposure (Figure 4C,D), in line with previous observations from human OA patients [42, 43]. Furthermore, ROS treatment also impacted the subcellular localization of SOX9. While SOX9 was predominantly localized within the cytoplasm of control cells, ROS treatment triggered a robust nuclear translocation at 1 h post‐treatment (Figure 4C,E). By 4 h, however, nuclear SOX9 localization was diminished, accompanied by an overall reduction in SOX9 abundance and a gradual redistribution back to the cytoplasm (Figure 4C,E). These observations suggest that acute oxidative stress initially alters SOX9 subcellular localization before the overall decline in SOX9 expression becomes predominant, potentially reflecting an early stress‐responsive regulatory mechanism. Nevertheless, at 4 h of acute ROS exposure, overall reduction in SOX9 expression and a gradual redistribution of the remaining SOX9 from the nucleus to the cytoplasm is observed, which may impair its transcriptional activity and contribute to the progressive loss of the chondrogenic phenotype. These results suggest that ROS‐mediated downregulation of SOX9 could impair transcription of key cartilage‐related genes, adversely affecting chondrocyte functionality and articular cartilage integrity.

Taken together, these findings demonstrate that the temporal impedance changes described in Section 2.3 coincide with independently validated molecular features of OA‐associated chondrocyte remodeling, including COL2 downregulation, COL1 upregulation, and dynamic alterations in SOX9 expression and intracellular localization. Notably, the transient nuclear translocation of SOX9 preceded its progressive decline, suggesting an early stress‐responsive adaptation before phenotypic deterioration became predominant. Collectively, these observations support the use of impedance profiling as a non‐invasive surrogate for longitudinal monitoring of OA‐associated phenotypic remodeling in vitro.

### 2.5 Bioelectrical Mapping Detects Matrix Mineralization‐Associated Remodeling During Chondrocyte Osteogenic Reprogramming

During long bone development and fracture repair, transient cartilage acts as a template for bone formation in a process called endochondral ossification, during which a subset of chondrocytes undergo de‐differentiation toward the osteogenic lineage [18]. Interestingly, an emerging school of thought advocates that this process is aberrantly activated during OA, accelerating cartilage calcification and osteochondral remodeling [16, 17]. Since oxidative stress induced COL2‐to‐COL1 isoform switching consistent with phenotypic remodeling, we next investigated whether the CMOS‐MEA platform could detect more extensive osteogenic reprogramming induced under defined osteogenic conditions. Compared to control cells, strong temporal increases in impedance were associated with osteogenic differentiation (Control: D0, Z = 0.386 ± 0.092 MΩ; D4, Z = 0.373 ± 0.096 MΩ; and D7, Z = 0.356 ± 0.087 MΩ; whereas OD: D0, Z = 0.391 ± 0.092 MΩ; D4, Z = 0.583 ± 0.151 MΩ; and D7, Z = 0.747 ± 0.147 MΩ) (Figure 5A,B). Immunostainings demonstrated that osteogenic differentiation led to gradual downregulation of COL2 and upregulation of COL1 protein expression at day 7 (Figure 5C). This molecular profile was consistent with osteogenic phenotypic reprogramming. Likewise, alizarin red staining, which stains calcium deposits, showed that osteogenic differentiation initiated matrix calcification (Figure 5D), further validating transdifferentiation of chondrocytes. Representative Hoechst stainings did not reveal major differences in cellularity between control and osteogenic conditions, suggesting that the pronounced impedance increase was not solely explained by overt differences in cell number. Thus, osteogenic induction resulted in increased impedance that coincided with osteogenic phenotypic reprogramming, extracellular matrix remodeling, and matrix mineralization. Because impedance represents an integrated biophysical readout, the relative contributions of cellular occupancy, cell‐electrode interactions, matrix deposition, and mineralization cannot be fully distinguished. The observed impedance increase should therefore be interpreted as a composite signature of osteogenic remodeling rather than a direct measure of any single biological process.

**FIGURE 5:**
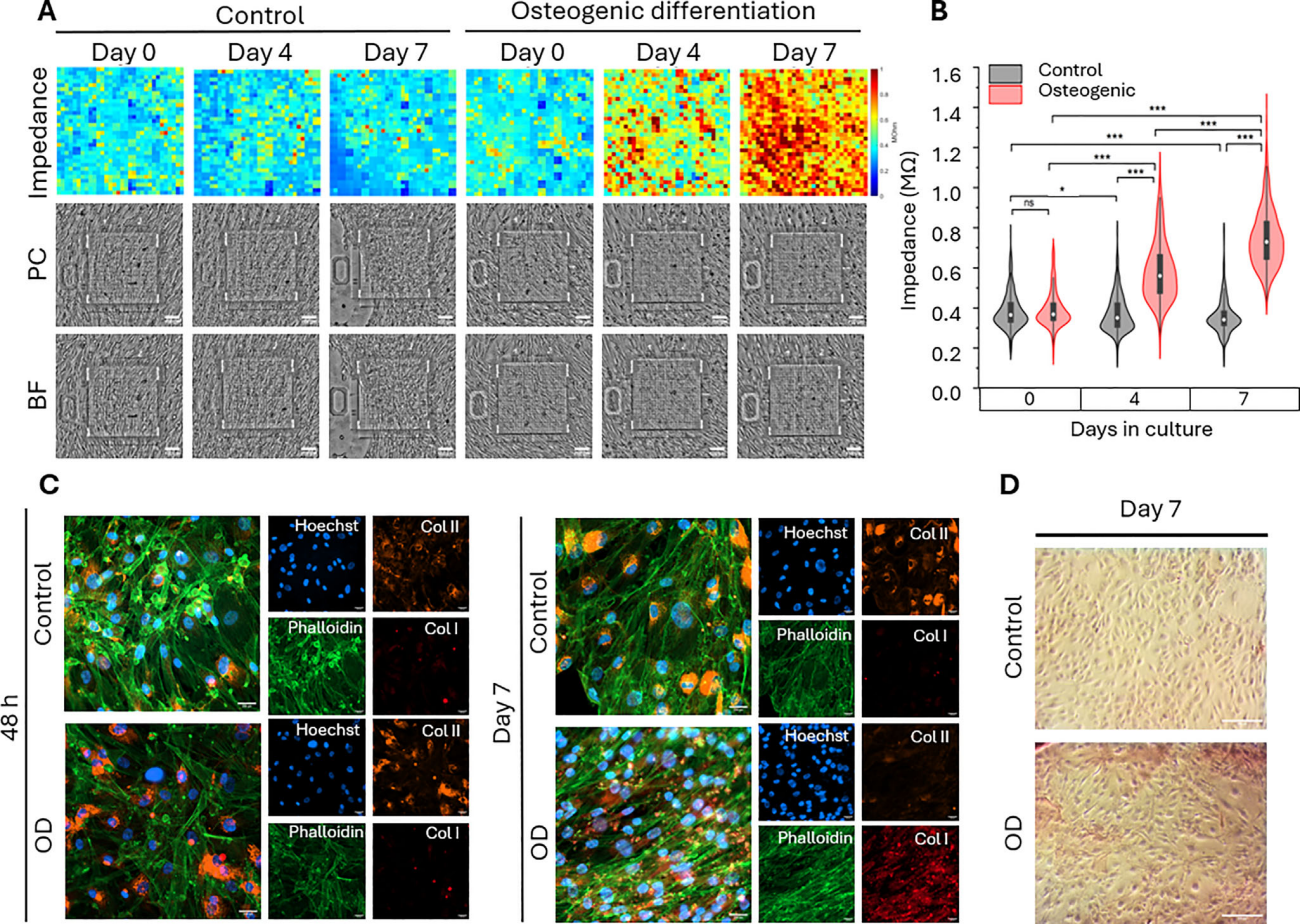
Bioelectrical signatures rise with osteogenic differentiation and matrix remodeling. Chondrocytes cultured on‐ and off‐chips were treated with either control media or osteogenic differentiation media for a period of 7 days. (A) Impedance heatmaps and corresponding phase contrast and brightfield images demonstrated temporal increases in impedance following osteogenic differentiation. Scale bars denote 100 µm. (B) Violin plot with nested box plots reveals increased impedance in response to osteogenic differentiation; *n* = 3; *N* = >1000. (C) Representative fluorescent images show COL2 downregulation and COL1 upregulation upon osteogenic differentiation. Hoechst 33342 was used to counter‐stain nuclei, whereas phalloidin was used for F‐actin staining. Pseudo‐colors for COL2, COL1, F‐actin, and Hoechst are amber, red, green, and blue, respectively. Scale bar denotes 50 µm; *n* = 3, *N* = >1000. (D) Alizarin red staining depicts the initiation of matrix mineralization following osteogenic differentiation. Scale bar represents 100 (A) and 200 (C‐D) µm; *n* = 3. PC, phase contrast; BF, brightfield. *P*‐values < 0.05, 0.01, and 0.001 are represented by *, **, and ***, respectively.

## 3 Discussion

Regulatory authorities and funding organizations are increasingly advocating the implementation of in vitro human model systems for fundamental research, pharmacological development, and efficacy evaluations to address ethical dilemmas and the inadequate translational relevance of animal models [20]. The inability of animal models to accurately mirror essential aspects of human physiology renders them ineffective in pinpointing targeted, disease‐modifying interventions. As a result, numerous prevalent disorders associated with the brain, heart, lungs, and musculoskeletal system that primarily relied on animal models still lack effective disease‐modifying therapies. In recent times, OoC models have facilitated the discovery of novel therapeutic targets for various ailments, such as autosomal recessive polycystic kidney disease and sepsis, which traditional static models failed to elucidate, underscoring the physiological relevance of OoC in advancing drug development initiatives [44, 45]. Nevertheless, the majority of the presently available in vitro systems designed to replicate human physiology exhibit two significant limitations: (1) they often adopt stringent reductionist designs that overlook key considerations regarding complex cell–cell and cell–ECM dynamics; and (2) they are devoid of non‐invasive dynamic monitoring capabilities equipped with spatial and single‐cell resolutions. Thus, in addition to biological relevance, equal emphasis must be placed on the identification and integration of dynamic monitoring technologies compatible with complex in vitro models. This is particularly essential for fibrotic diseases, such OA, which are driven by spatiotemporally dysregulated cell communication networks between multi‐tissue compartments, necessitating not merely dynamic monitoring but also spatial and single‐cell resolution [12, 46]. This technological bottleneck inspired the genesis of the present study, which concentrated on exploring the applicability of electrical impedance utilizing a high‐density CMOS MEA platform for dynamic single‐cell monitoring of OA‐associated cellular alterations. Addressing this technological gap is equally valuable in well‐controlled cellular models, where dynamic cellular responses can be rigorously characterized, and in increasingly sophisticated multicellular MPSs, where longitudinal monitoring is expected to provide unprecedented insight into evolving cell‐state transitions and tissue remodeling.

A major finding of the present study is that high‐density CMOS‐MEA impedance profiling provides a continuous, non‐invasive electrical readout of chondrocyte state that evolves in parallel with OA‐relevant phenotypic remodeling. Rather than reflecting a single biological process, the impedance signal integrates multiple physical and biological properties of the cell culture, including cell coverage, cell–substrate interactions, ECM remodeling, morphology, and viability. CMOS MEA‐based impedance profiling accurately mapped chondrocyte proliferation and migration kinetics, which mimic the in vivo emergence of highly proliferative and migratory phenotypes in response to cartilage injury or mechanical trauma [35, 47, 48]. Notably, our results showed that migration‐based substrate attachment dynamics led to localized impedance reductions in areas adjacent to the wound edges, suggesting that migration‐associated focal adhesion turnover might be monitored through impedance changes. Additional experiments utilizing EDTA substantiated that alterations in cell adhesion were directly correlated with impedance fluctuations, highlighting the added potential of electrical impedance to evaluate cell adhesion dynamics. Furthermore, impedance changes were associated with OA‐induced phenotypic shifts in chondrocytes. These shifts encompassed not only morphological changes [7, 49, 50], apoptosis [15, 51, 52], and collagen switching [53], which are observed during the early stages of OA development, but also matrix mineralization [54]. and de‐differentiation [17], which are characteristic of advanced OA. This validates the capacity of impedance recording to deliver biologically pertinent insights throughout various stages of the disease, thereby providing a comprehensive understanding of stage‐appropriate chondrocyte behavior in OA. Additionally, our findings broaden the current comprehension of OA pathophysiology by demonstrating that the increased nuclear translocation of SOX9 in response to oxidative stress serves as an early compensatory mechanism employed by chondrocytes for cellular survival. This mechanism is supported by observations in human OA cartilage, where SOX9 expression was found to be upregulated during early stages of the disease but declined in advanced stages, hinting at a transient pro‐survival response aimed at restoring matrix integrity, potentially through SOX9‐dependent ADAMTS inhibition [42].

Our findings demonstrate that CMOS MEA‐based impedance recording provides functional quantitative insights into electrochemical changes associated with a multitude of biological processes that are disrupted during fibrosis and OA, including proliferation, migration, adhesion, stress response, and de‐differentiation. Currently, the majority of commercially available dynamic monitoring instruments employ biosensors capable of detecting a finite number of changes in oxygen, pH, or soluble metabolites/protein biomarkers [22], which serve as surrogates for cellular health, representing homogenous changes in the entire cell population. Conversely, impedance monitoring with our system provides a direct assessment at the level of individual cells. In line with our results, a few previous studies have documented impedance alterations in relation to proliferation and adhesion in other cell types, such as fibroblasts and epithelial cells [27, 28, 55]. Although our investigation concentrated on OA as a representative disease, our technology is relevant for fundamental and translational studies across a diverse spectrum of human pathophysiologies. While its non‐invasive, label‐free, and dynamic monitoring capabilities are highly significant, its spatial and single‐cell resolution features are particularly noteworthy, presenting opportunities to explore the impacts of cellular heterogeneity and biological variability. The integration of the CMOS MEA platform with physiologically relevant tissue or organ MPSs presents a promising scalable and high‐throughput methodology for assessing disease mechanisms in vitro and for screening potential human disease‐modifying therapies.

While the present study establishes the feasibility of high‐density CMOS‐MEA impedance profiling for longitudinal monitoring of OA‐relevant chondrocyte remodeling, several opportunities remain for further development. The presented work used static concentrations of ROS and DMSO, which fail to capture the dynamic oxidative fluctuations encountered in vivo. While our study focuses on acute ROS exposure that recapitulates the microenvironment following traumatic joint injuries, it would also be interesting to explore the impedance and cellular dynamics in response to chronic low‐grade oxidative stress. This could be achieved by providing low concentration, oscillatory, or pulsed ROS exposure, mimicking the chronic OA joint state. Likewise, the incorporation of variable ROS exposures could yield more physiologically pertinent insights. An important consideration is that impedance represents a composite biophysical readout whose interpretation depends on biological context. Rather than reporting a single cellular process, impedance integrates multiple parameters, including cell density, cell–substrate interactions, morphology, viability, differentiation state, extracellular matrix deposition, and potentially cell‐cycle‐dependent changes. Consistent with this, impedance primarily reflected increasing cellular coverage during early culture growth, changes in cell distribution and adhesion during wound closure and EDTA treatment, combined effects of morphology, attachment, and viability during oxidative stress, and matrix remodeling and mineralization during osteogenic induction. Therefore, the value of the CMOS‐MEA platform lies not in assigning impedance to a single biological mechanism, but in providing a continuous, spatially resolved functional readout that can be interpreted in conjunction with orthogonal validation assays. Accordingly, impedance measurements were complemented by microscopy, immunocytochemistry, viability assays, and matrix analyses throughout this study. Once the principal biological contributors to impedance have been defined for a given system, longitudinal impedance monitoring can reduce reliance on repeated destructive endpoint analyses, enabling dynamic follow‐up using a limited set of validation assays. Future integration with real‐time imaging, reporter systems, computational modeling, and machine‐learning‐based pattern recognition may further enhance biological interpretation and facilitate the extraction of mechanistic insights from complex impedance signatures.

Finally, although the present work employed a well‐controlled two‐dimensional primary chondrocyte model, this should be viewed as an essential technology‐validation step rather than the intended endpoint of the platform. Impedance‐based measurements are intrinsically influenced by biological, electrical, and physicochemical parameters; establishing a rigorous understanding of these relationships in a simplified system is therefore a necessary prerequisite before extending the technology to more physiologically complex models, where additional factors such as matrix composition, diffusion gradients, mechanical properties, and heterogeneous tissue architecture introduce further complexity to data interpretation. Hence, our experiments serve as an essential foundational step toward developing physiologically relevant high‐density MEA 3D models in future work. Moreover, our work successfully resolves several key technical considerations necessary for successful integration into 3D tissues: robust, long‐term biocompatibility of the material used to encapsulate the electronics; reliable post hoc alignment of microelectrodes with dynamic, growing cultures; and non‐destructive, real‐time, longitudinal read‐outs of cell physiology, reducing the reliance on destructive end‐point assays.

Nevertheless, integrating CMOS MEA technology into OoC or MPS platforms remains fraught with significant technical challenges. Within an MPS environment, maintaining tissue viability in close proximity to embedded electronics requires stringent control of heat dissipation and electrochemical by‐products generated during long‐term operation. Sterility and leak‐proof incorporation of microfluidic systems become increasingly complex when interfacing with high‐density electronic architectures. Additionally, electrical routing must be designed so that it does not obstruct perfusion channels or interfere with optical access needed for high‐resolution imaging. For compatibility with 3D organoids or hydrogel‐based MPSs, mesh MEAs represent a promising strategy [56]. However, their implementation introduces further challenges, including ensuring long‐term mechanical stability of ultrathin, flexible meshes under continuous fluid flow or tissue contraction, and matching the mechanical stiffness of the mesh to soft hydrogel matrices to avoid imposing unintended mechanical cues on the developing tissue. If addressed successfully, such systems would enable true 3D impedance measurements and provide more physiologically relevant insight into tissue‐level dynamics. Finally, scalability remains a critical consideration for pharmaceutical applications. This includes reducing per‐chip manufacturing costs while preserving the high electrode density required for real‐time electrophysiology and impedance mapping, as well as ensuring compatibility with automated liquid‐handling systems, robotic screening platforms, and standardized multi‐well or multi‐chip formats (e.g., SBS‐compliant plates) to allow efficient parallelization.

## 4 Conclusion

This study reports the utility of CMOS MEA‐based impedance profiling for non‐invasive, label‐free, dynamic assessment of diverse biological processes at spatial and single‐cell resolution. Using OA as a representative disease model, the platform successfully tracked cell proliferation, morphological changes, ECM alterations, migration, adhesion dynamics, stress responses, and de‐differentiation, which constitute processes that are commonly dysregulated in fibrosis, chronic inflammation, tissue regeneration, and cancer. Importantly, impedance represents a composite, context‐dependent biophysical readout influenced by cell occupancy, morphology, cell–substrate interactions, viability, ECM deposition, and mineralization; it cannot independently distinguish the contribution of each process and therefore requires orthogonal biological validation for mechanistic interpretation. With this consideration, the findings validate CMOS MEA technology as a powerful tool for advancing basic research, therapeutic screening, and efficacy testing in human OA pathobiology, with broader applicability to diverse human diseases. The CMOS MEA platform thus provides robust non‐invasive monitoring functionality for integration with in vitro models tailored to dynamically map cellular alterations at spatial and single‐cell resolution, enabling technology‐driven biological breakthroughs to guide mechanism‐driven drug discovery.

## 5 Materials and Methods

### 5.1 General Cell Culture

Primary human articular cartilage‐derived chondrocytes (cat. #P10970) were procured from Innoprot (Bizkaia, Spain) and cultured using commercially available media kits (cat. #P60137, Innoprot). All procedures related to thawing, maintaining, and subculturing were performed according to the supplier's specific instructions. Briefly, for routine sub‐culturing, cells were seeded at a density of 4,000 cells/cm^2^ in T75 flasks that had been coated overnight with poly‐L‐lysin (2 µg/cm^2^) (Innoprot, cat. #PLL) and were cultured in a humidified incubator set at 37°C and 5% CO_2_. Cells were passaged at 70%–80% confluency and seeded for experiments, as described in later sections. All experiments were conducted between passages P3–7. For fluorescent tracking, cells were incubated with 1 µM Celltracker Green CMFDA (cat. #C2925, Thermo Fisher, Massachusetts, USA) for 40 min, followed by 10 min incubation with 1 µg/mL Hoechst 33342 (cat. #H3570, Thermo Fisher). The stained cells were imaged at specified timepoints using Zeiss Axio Examiner.Z1 (Zeiss, Jena, Germany).

### 5.2 CMOS MEA Design, Fabrication, and Post‐Processing

CMOS MEA chips are fabricated using 0.13 µm CMOS technology and a six‐metal‐layer aluminum back‐end‐of‐line (BEOL) stack. The chips are subjected to post‐processing steps, such as passivation, via opening, electrode deposition, and bond pad opening. The cell culture area harboring the active sensors spans a surface area of 0.5 cm^2^ and consists of an array of 16 384 electrodes connected to 4096 pixels and divided into 16 separate clusters (Figure S1A). Each cluster, thus, has 256 pixels and 1024 electrodes. Eight additional pixels in each cluster are used as references and are connected to large reference electrodes (235 × 50 µm^2^) placed around the clusters. The recording pixels are connected to 1024 recording channels and 64 stimulation units. With these, the CMOS MEA chips are equipped with six cell‐interfacing modalities: (1) extracellular recording (in 1024 simultaneous sites); (2) intracellular recording (1024 sites); (3) constant voltage stimulation for controlled electrode potential (4096 sites); (4) constant‐current stimulation for controlled charge delivery (64 sites); (5) fast impedance monitoring at a fixed frequency (1024 sites); and (6) impedance spectroscopy in a frequency range of 10 Hz–1 MHz (64 sites). These multi‐modal electrophysiological monitoring capabilities allow for a wide range of applications, improving the overall functionality of the chips.

### 5.3 Cleaning and Sterilization of CMOS MEA Chips

Prior to seeding cells on chips and at the end of each experiment, chips were incubated with 1% Terg‐A‐zyme (Cat. # Z742918, Sigma‐Aldrich, Missouri, USA) overnight at 37°C, followed by thorough rinsing with high purity water (HPW) to remove cellular debris and detergent residue. Thereafter, the chips were sterilized with 70% ethanol for 30 min, rinsed with HPW, and dried out for 30 min. The chips were stored at this stage or coated with poly‐L‐lysine (PLL) for cell seeding.

### 5.4 Cell Seeding on the CMOS MEA System

Chips were cleaned and sterilized as mentioned above and then coated with PLL (2 µg/cm^2^; Cat. #PLL, Innoprot) one day prior to seeding to enhance cell attachment. For cell seeding, chondrocytes (P3–7) were harvested from 70% confluent T‐75 flasks after repeated PBS wash followed by incubation in 5 mL of 0.025% trypsin‐EDTA (cat. #25200072, Thermo Fisher) for 3 min. The trypsin was quenched by adding 5 mL of complete growth media, and the resultant cell suspension was centrifuged at 1200 rpm for 5 min. Supernatant was discarded, and the pellet was resuspended in 1 mL of complete media. Live cell count was determined using a trypan blue exclusion assay in a hemocytometer, and chondrocytes were seeded at a cell density of 30 000 cells per cm^2^ (15 000 cells per chip). To investigate the contribution of cell seeding density to impedance, cells were seeded at 5k/chip, 10k/chip, 15k/chip, and 20k/chip. For all experiments, media was changed one day post‐seeding and every 2 days thereafter. Cells cultures were maintained in an incubator with the following conditions: 37°C, 96% humidity and 5% CO_2_. For every chip, parallel controls were created by attaching an adhesive acrylic insert of the same dimension (surface area: 0.5 cm^2^ [Cat. # 80209, Ibidi, Gräfelfing, Germany]) to a 60 mm petri dish, seeded, and treated identically.

### 5.5 Fixed‐Frequency Impedance Measurements and Data Analysis

Impedance measurements were conducted on CMOS MEA chips. The chips were connected to an IMEAbox via a USB‐C cable, with data transferred to a PC using a USB‐3 cable. Multi‐channel impedance recordings were performed using internal software called ‘the Sparrow app’, applying an AC of 1 nA at 1 kHz. Before seeding, impedance measurements were performed on PLL‐coated chips containing media alone to establish blanks. Impedance measurements of all the wells per chip were recorded at the specified timepoints. Recorded impedance values per electrode were exported as csv files, then decoded and analyzed using MATLAB R2024a. A custom MATLAB script generated 2D color‐coded spatial impedance heatmaps. The script mapped each electrode to its corresponding physical location within the well or cluster, essentially rendering a spatial single‐cell resolution impedance map. For spatial impedance heatmaps, impedance data were scaled from 0 to 1 MΩ to enhance visual contrast and highlight spatial variations within the biologically relevant impedance range. Raw impedance data were plotted as violin plots with nested box plots to clearly demonstrate the entirety of the data distribution and used for statistical analysis. Within the nested box plot, the white circle denotes the median, the box represents the interquartile range, and the whiskers indicate 1.5 × IQR. To measure impedance per cell, the exact occupancy of the cell on the electrode‐dense active area was identified from CellTracker Green‐stained fluorescent and brightfield images, based on the fiducial marks present on the chip and around each active area. Each active region of the chip consists of 1,024 electrodes with 15 µm pitch and 8 µm electrode diameter. Given that adherent mammalian cells typically range in size from 18–30 µm, each cell is expected to cover ∼1–4 electrodes. Impedance per cell was calculated as a weighted mean of the impedance values from the electrodes occupied by that cell. Datapoints/electrodes were omitted in case of conflict with cell attribution.

### 5.6 Cell Proliferation

Chondrocytes were seeded at a density of 30 000 cells/cm^2^ onto the chips and incubated at 37 °C under 96% humidity and 5% CO_2_. A media change was performed one day post‐seeding and subsequently every 2 days. Impedance scans were recorded at 1 kHz and 1 nA every 2 days, in parallel with optical analyses. Brightfield imaging was conducted using a Keyence VHX‐7000 microscope, and fluorescence imaging was performed with a Zeiss Axio Examiner. Concurrently, a PrestoBlue assay was used to assess cell viability and proliferation. Briefly, cells were incubated with PrestoBlue reagent diluted 1:10 in culture medium for 1 h at 37 °C in the dark. After incubation, 100 µL of the reagent was collected, and fluorescence was measured using a Tecan plate reader (Infinite M200 Pro, Tecan, Zürich, Switzerland) with excitation and emission wavelengths of 560 and 590 nm, respectively. Baseline fluorescence from blank wells was subtracted, and the resulting values were plotted. For all on‐chip experiments, parallel control cultures were seeded in Ibidi inserts placed on standard culture dishes and treated identically to confirm that the growth characteristics were not altered by the chip environment.

### 5.7 Picrosirius Red Assay

Collagen deposition was assessed using the Picrosirius Red staining kit (Abcam, ab150681) following the manufacturer's protocol. Briefly, samples were fixed in 4% paraformaldehyde for 15 min, rinsed with PBS, and incubated in Picrosirius Red solution for 60 min at RT. After staining, samples were washed twice with 0.5% acetic acid to remove unbound dye, dehydrated through graded ethanol, and mounted in DPX. Imaging was performed on a Zeiss Axio Imager microscope (AxioCam 208) under both brightfield and polarized light to visualize total collagen and birefringent collagen fibers, respectively. Raw CZI files were processed and analyzed in ImageJ/Fiji. For brightfield images, the red‐stained collagen signal was thresholded and quantified, with values normalized to the number of cells per field of view. For polarized light images, birefringent collagen was quantified by measuring the integrated intensity within thresholded collagen‐positive regions.

### 5.8 ROS Stimulation

Chondrocytes were seeded at a density of 30 000 cells/cm^2^ onto chips and Ibidi inserts as technical controls. Once the monolayer was 70%–80% confluent, the cells were treated with 1500 µM hydrogen peroxide (H_2_O_2_, Cat. #10188601, Honeywell, North Carolina, USA) for 1 and 4 h. Non‐stimulated cells and cells treated for 4 h with 5% DMSO (Cat. # D12345, Thermo Fisher Scientific) served as negative and positive controls, respectively. For impedance recording, an initial scan was recorded before treating the cells with H_2_O_2_ and thereafter at every 10 min for a period of 4 h. For morphological analysis, brightfield and fluorescent images were captured using Zeiss Axio Examiner with 5 X and 10 X objectives at 0, 1 and 4 h, respectively. To monitor alterations in protein expression, technical replicates seeded on Ibidi inserts were fixed at 1‐ and 4 h post‐ROS treatment and subjected to immunocytochemical analysis of the specified proteins.

### 5.9 Measurement of Cell Detachment and Reattachment Kinetics

Chondrocytes were seeded at a density of 20 000 cells/cm^2^ onto chips and cultured for 48 h. After an initial impedance scan and fluorescent imaging, cells were incubated with 5 mM EDTA for a period of 2.5 h, and impedance scans were taken every 15 min. Thereafter, EDTA was removed, and cells were allowed to recover in standard complete media. Immediately after EDTA removal, fluorescent images were captured to determine morphological changes. Recovery was monitored with impedance scans every 15 min for a period of 8 h.

### 5.10 Scratch Assay

A scratch assay was performed to evaluate the migration capabilities of chondrocytes, a crucial step in tissue regeneration, using CMOS MEA chips. Briefly, a manual scratch was created using a sterile P1000 tip in a confluent monolayer of chondrocytes cultured on CMOS MEA chips. The chip comprises 16 wells, arranged in a 4 x 4 matrix. The scratch was sketched to cover the distance between the centers of the wells in lanes 2 and 3, measuring approximately 1500 µm. Cell migration within the wound area was monitored electrically every hour for a period of 48 h using EIS measurements. Parallel scratches made on chondrocyte monolayers on Ibidi inserts were monitored visually using Zeiss Primo Vert microscope at 10X and 20X magnification.

### 5.11 Differentiation Assay

Briefly, chondrocytes cultured on chips and in control inserts were treated with either standard chondrocyte media (control) or osteogenic differentiation (OD) media. The OD media consisted of DMEM high glucose supplemented with 100 µg/mL ascorbic acid, 100 nM dexamethasone, 10 mM β‐glycerophosphate, 5% fetal bovine serum (FBS), 1% Glutamax, and 1% penicillin/streptomycin. Media was changed every 2 days. Impedance scans and imaging data were collected at 0‐, 4‐, and 7‐day post‐treatment. Parallel cultures seeded on Ibidi inserts and subjected to identical treatments were fixed on day 7 with 4% paraformaldehyde (PFA, Cat. # J19943.K2, Thermo Scientific) for 15 min at room temperature. Osteogenic differentiation was assessed by immunostaining for collagen I, collagen II, and phalloidin. Alizaren red staining was performed on parallel inserts to assess matrix mineralization. Briefly, PFA‐fixed samples were washed three times with distilled water. Cells were then incubated with 2% Alizarin Red S solution (pH 4.2; Cat. #A5533, Sigma Aldrich) for 20 min at room temperature with gentle agitation. Excess stain was removed by washing the samples thoroughly with distilled water until the wash solution was clear. Samples were air‐dried prior to imaging using Zeiss Primo Vert microscope at 5X magnification.

### 5.12 Immunostaining

Immunocytochemistry was performed to assess the expression of key differentiation markers, including osteocalcin, collagen type I, collagen type II, and SOX9. At specified time points, cells were fixed with 4% PFA for 15 min at room temperature. Cells were then permeabilized with 0.1% Triton X‐100 (Cat. #9036195, Sigma Aldrich) for 15 min, followed by blocking of non‐specific binding sites with 5% bovine serum albumin (BSA; Cat. #05470‐25G, Sigma Aldrich) for 1 h. Primary antibodies (listed in Table 1) were incubated overnight at room temperature. After washing thrice with Dulbecco's phosphate‐buffered saline (DPBS) containing 0.1% Triton X‐100, cells were incubated for 1 h with the corresponding secondary antibodies (Table 1). The cytoskeleton and nuclei were counterstained with phalloidin conjugated to Alexa Fluor 488 and Hoechst 33342, respectively.

**TABLE 1:** Primary and secondary antibodies.

Molecular target	Company	Catalog no.	Concentration/Dilution
Primary antibodies			
Sox9	Novus biologicals	NBP1‐85551	1:200
Collagen I	Novus biologicals	NBP1‐30054	1:100
Collagen II	Novus biologicals	NB600‐844	1:100
Osteocalcin	Novus biologicals	MAB1419	25 µg/mL
Phalloidin (F‐actin)	Invitrogen	A12379	0.165 µM

Secondary antibodies and dyes			
Anti‐rabbit Alexa Fluor 488	Invitrogen	A21379	2.5%
Anti‐rabbit Alexa Fluor 568	Thermo Fisher	A‐11011	2.5%
Anti‐mouse Alexa Fluor 647	Thermo Fisher	MG121	2.5%
Hoechst 33342	Thermo Fisher	H3570	2.5 µg/mL

Photomicrographs were captured in Hank's balanced salt solution (HBSS) using a Zeiss Axio Examiner microscope equipped with an ORCA Flash camera. Image processing and analysis were conducted using ZEN software (v3.9, Zeiss) or ImageJ (Fiji, NIH). Images were uniformly processed to subtract background fluorescence. Cell boundaries were delineated as regions of interest (ROIs) using either thresholding or Cellpose (v3.1.1.2). Quantification of cell area, mean fluorescence intensity, and integrated fluorescence intensity was performed on a per‐cell basis. A minimum of 100 cells were analyzed for each condition.

### 5.13 Statistics

Data were processed using MATLAB (R2024a), while data visualization and statistical analyses were performed using Origin (2024) and R. Impedance data were visualized as violin plots with overlaid boxplot elements. In these plots, black boxes represent the interquartile range (25%–75%), whiskers indicate 1.5× IQR, and white circles denote the median. To account for the non‐independent nature of repeated measurements collected from the same electrodes, statistical significance for impedance datasets was assessed using repeated‐measures ANOVA (rmANOVA) and linear mixed‐effects models (LMMs). For rmANOVA, *p*‐values were adjusted using the Greenhouse–Geisser correction due to violation of the sphericity assumption. Post‐hoc pairwise comparisons were performed using the Friedman test, a non‐parametric approach suitable for non‐normally distributed impedance measurements, with Bonferroni correction applied to account for multiple testing. For LMMs, expected marginal means were computed for each timepoint, and pairwise comparisons were conducted using the Kenward–Roger method to calculate fractional degrees of freedom, with *p*‐values adjusted using Tukey's method (Table S1‐6). For impedance heatmaps, data were scaled to a range of 0–1 MΩ, with blue representing 0 MΩ and red representing 1 MΩ. For fluorescence intensity measurements and other assays, statistical significance was evaluated using unpaired Student's *t*‐tests or one‐way ANOVA with Tukey's or Bonferroni post‐hoc tests, as appropriate. A minimum of 100 individual cells were analyzed per condition. Data are presented as mean ± standard deviation (SD); ‘*n*’ denotes the number of biological replicates, while ‘*N*’ indicates the number of cells or electrodes analyzed per experiment. All experiments were conducted in triplicate unless otherwise indicated, with exact sample sizes (*n*) provided in the figure legends. Statistical significance is indicated as *, **, and *** for *p* < 0.05, *p* < 0.01, and *p* < 0.001, respectively.

## Conflicts of Interest

The authors declare no conflicts of interest.

## Supporting information




**Supporting File 1**: adhm71837‐sup‐0001‐SuppMat.docx.

**Supporting File 2**: adhm71837‐sup‐0002‐movie S1.avi.

## Data Availability

The data that support the findings of this study are available in the supporting information of this article.
