# Supplementary material for: Single‐Cell Resolution Mapping of Osteoarthritis‐Associated Chondrocyte Dynamics Using a High‐Density CMOS MEA Platform
Source: Adv Healthc Mater. 2026 Oct 14;15(42):e04115. doi: 10.1002/adhm.202504115 (PMC13768201; doi:10.1002/adhm.202504115)
Supplement: Supplementary file 1 [file ADHM-15-0-s002.docx]

Supporting Information

**Single-cell resolution mapping of osteoarthritis-associated chondrocyte dynamics using a high-density CMOS MEA platform**

*Neha Deshpande^1^, Pauline Coquart^1,2^, Bram Theuns ^1^, Susanne Cremery^1^, Jaime Rodolfo V. Garcia III^1^, Julien Verplanken^1^, Dennis Lambrechts^1^, Dries Braeken^1^, Liesbet Lagae^1,2^, and Johanna Bolander^1,3,4^**

^1^imec, Kapeldreef 75, 3001 Leuven, Belgium

^2^Department of Physics and Astronomy, KU Leuven, Celestijnenlaan 200d, 3001, Leuven, Belgium

^3^Berlin Institute of Health at Charité - Universitätsmedizin, BIH Center for Regenerative Therapies, Berlin, Germany

^4^Berlin Institute of Health at Charité - Universitätsmedizin, Julius Wolff Institute, Berlin, Germany.
*Corresponding author: E-mail: [johanna.bolander@imec.be](mailto:johanna.bolander@imec.be); [johanna.bolander@bih-charite.de](mailto:johanna.bolander@bih-charite.de)

**Supplementary Figure S1: CMOS MEA chip layout and impedance data distribution.
(A)** Leftmost, photograph of an active CMOS MEA chip with the cell culture area marked in yellow; middle, the 5 × 5 mm^2^ cell culture area of the chip depicts the 16 active areas, representing the high-density microelectrode clusters; rightmost, one 480 × 480 µm^2^ active area comprising evenly spaced 1,024 electrodes. **(B)** Violin plot depicts no change in base impedance of the electrodes after PLL-coating. **(C)** Additional panels from Figure 1A for Hoechst, CellTracker Green, brightfield (BF), and merged image are presented. **(D)** Seeding densities were validated using PrestoBlue cell viability assay. **(E)** Bode plot representing a frequency sweep demonstrates a clear separation between the impedance measurements of empty electrodes and chondrocyte-covered electrodes, particularly at 1 kHz and higher frequencies, across multiple days of culture. **(F)** Binned density histogram of mean impedance per cell (MΩ) display a gamma distribution, reflecting a normal distribution with a slight positive skew. All experiments were conducted in triplicates, with >800 cells and >1000 electrodes. P-values <0.05, 0.01, and 0.001 are represented by *, **, and ***, respectively.

**Supplementary Figure S2: Impedance changes capture cell-substrate adhesion dynamics.**

Chondrocytes seeded onto CMOS MEA chips were treated with 5 mM EDTA for 2.5 h. Thereafter, EDTA was removed, and cells were allowed to recover. **(A)** Brightfield (BF), phase contrast (PC), and fluorescence images before and after EDTA treatment are depicted. Scale bars denote 100 µm. **(B)** Heatmaps depict time-dependent impedance decrease upon EDTA treatment and progressive increase following recovery, reflecting cell detachment and reattachment kinetics, respectively. **(C)** Violin plots depict temporal changes in impedance in response to cell attachment and reattachment. All experiments were conducted in triplicates, with >1000 electrodes. P-values <0.05, 0.01, and 0.001 are represented by *, **, and ***, respectively. Statistics for comparisons between consecutive and representative time points are displayed in the graph. Comparison between all other time points are available in Supplementary Table S5.

**Supplementary Figure S3: DMSO treatment reduces cellular impedance.**

Chondrocytes seeded onto CMOS MEA chips were treated with 5% DMSO for 4 h. **(A)** Brightfield (BF), phase contrast (PC), and fluorescence images before and after DMSO treatment are depicted. Scale bars denote 100 µm. **(B)** Spatial impedance heatmaps depict time-dependent impedance decrease upon DMSO treatment. **(C)** Violin plots with overlaid box plots depict temporal changes in impedance in response to DMSO treatment. Statistics for comparisons between consecutive and representative time points are displayed in the graph. Comparison between all other time points are available in Supplementary Table S6. n = 3; N > 1000. **(D)** Parallel live dead assay reveals DMSO-mediated cell death and detachment, n = 3; N = 10-20 microscopic 10X images. P-values <0.05, 0.01, and 0.001 are represented by *, **, and ***, respectively.

**Supplementary Figure S4: ROS treatment alters cell morphology and reduces cell impedance.**

**(A)** Phase contrast microscopic images of chondrocytes treated with varying concentrations of H_2_O_2_ (ROS) for 4 h. Box plots reveal **(B)** area and **(C)** aspect ratio alterations in chondrocytes in response to 1500 µM ROS at 1 and 4 h; n = 3, N > 2000 cells. **(D)** Bode plot representing a frequency sweep depicts reduced impedance of chondrocytes in response to 150 µM ROS for 24 h. P-values <0.05, 0.01, and 0.001 are represented by *, **, and ***, respectively.

**Supplementary Video S1:**

Video depicts spatial impedance heatmaps of chondrocytes treated with 5% DMSO for 4 h recorded at every 15 min interval, presented in Figure S3A–C. A wave of decreasing impedance is noticed from the top right corner towards the bottom left corner, reflecting the spatial movement of cells seen in Figure S3A.

**Supplementary Table S1:** Statistical results from linear mixed-effects model (LMM) for Figure 1E

| **Figure 1E** |  |  |  |  |  |  |
| --- | --- | --- | --- | --- | --- | --- |
| **Contrast** | **Estimate** | **SE** | **df** | **t.ratio** | **p.value** | **significance** |
| Days1 - Days3 | 0.02086 | 0.00697 | 6138 | 2.99460 | 4.38E-02 | * |
| Days1 - Days5 | -0.09413 | 0.00697 | 6138 | -13.51333 | 0.00E+00 | *** |
| Days1 - Days7 | 0.00483 | 0.00697 | 6138 | 0.69316 | 9.93E-01 | ns |
| Days1 - Days9 | -0.04814 | 0.00697 | 6138 | -6.91049 | 1.07E-10 | *** |
| Days1 - Days11 | -0.12941 | 0.00697 | 6138 | -18.57721 | 0.00E+00 | *** |
| Days1 - Days13 | -0.10064 | 0.00697 | 6138 | -14.44750 | 0.00E+00 | *** |
| Days3 - Days5 | -0.11499 | 0.00697 | 6138 | -16.50793 | 0.00E+00 | *** |
| Days3 - Days7 | -0.01603 | 0.00697 | 6138 | -2.30143 | 2.43E-01 | ns |
| Days3 - Days9 | -0.06900 | 0.00697 | 6138 | -9.90509 | 0.00E+00 | *** |
| Days3 - Days11 | -0.15027 | 0.00697 | 6138 | -21.57181 | 0.00E+00 | *** |
| Days3 - Days13 | -0.12150 | 0.00697 | 6138 | -17.44209 | 0.00E+00 | *** |
| Days5 - Days7 | 0.09896 | 0.00697 | 6138 | 14.20649 | 0.00E+00 | *** |
| Days5 - Days9 | 0.04600 | 0.00697 | 6138 | 6.60284 | 9.13E-10 | *** |
| Days5 - Days11 | -0.03527 | 0.00697 | 6138 | -5.06388 | 8.77E-06 | *** |
| Days5 - Days13 | -0.00651 | 0.00697 | 6138 | -0.93417 | 9.67E-01 | ns |
| Days7 - Days9 | -0.05297 | 0.00697 | 6138 | -7.60365 | 0.00E+00 | *** |
| Days7 - Days11 | -0.13424 | 0.00697 | 6138 | -19.27037 | 0.00E+00 | *** |
| Days7 - Days13 | -0.10547 | 0.00697 | 6138 | -15.14066 | 0.00E+00 | *** |
| Days9 - Days11 | -0.08127 | 0.00697 | 6138 | -11.66672 | 0.00E+00 | *** |
| Days9 - Days13 | -0.05250 | 0.00697 | 6138 | -7.53701 | 0.00E+00 | *** |
| Days11 - Days13 | 0.02877 | 0.00697 | 6138 | 4.12971 | 7.28E-04 | *** |

**Supplementary Table S2:** Statistical results from linear mixed-effects model (LMM) for Figure 2B–D

| **Figure 2B** |  |  |  |  |  |  |
| --- | --- | --- | --- | --- | --- | --- |
| **Contrast** | **Estimate** | **SE** | **df** | **t.ratio** | **p.value** | **Significance** |
| (Hours-1) - Hours0 | 0.11297 | 0.00418 | 5964 | 27.04524 | 1.96E-12 | *** |
| (Hours-1) - Hours6 | 0.16297 | 0.00418 | 5964 | 39.01572 | 1.96E-12 | *** |
| (Hours-1) - Hours12 | 0.17731 | 0.00418 | 5964 | 42.44697 | 1.96E-12 | *** |
| (Hours-1) - Hours18 | 0.20690 | 0.00418 | 5964 | 49.53099 | 1.96E-12 | *** |
| (Hours-1) - Hours24 | 0.13958 | 0.00418 | 5964 | 33.41526 | 1.96E-12 | *** |
| (Hours-1) - Hours48 | 0.06797 | 0.00418 | 5964 | 16.27133 | 1.96E-12 | *** |
| Hours0 - Hours6 | 0.05000 | 0.00418 | 5964 | 11.97048 | 1.96E-12 | *** |
| Hours0 - Hours12 | 0.06433 | 0.00418 | 5964 | 15.40173 | 1.96E-12 | *** |
| Hours0 - Hours18 | 0.09393 | 0.00418 | 5964 | 22.48575 | 1.96E-12 | *** |
| Hours0 - Hours24 | 0.02661 | 0.00418 | 5964 | 6.37002 | 4.26E-09 | *** |
| Hours0 - Hours48 | -0.04500 | 0.00418 | 5964 | -10.77390 | 2.03E-12 | *** |
| Hours6 - Hours12 | 0.01433 | 0.00418 | 5964 | 3.43125 | 1.08E-02 | * |
| Hours6 - Hours18 | 0.04392 | 0.00418 | 5964 | 10.51527 | 2.03E-12 | *** |
| Hours6 - Hours24 | -0.02339 | 0.00418 | 5964 | -5.60045 | 4.67E-07 | *** |
| Hours6 - Hours48 | -0.09501 | 0.00418 | 5964 | -22.74438 | 1.96E-12 | *** |
| Hours12 - Hours18 | 0.02959 | 0.00418 | 5964 | 7.08402 | 3.48E-11 | *** |
| Hours12 - Hours24 | -0.03773 | 0.00418 | 5964 | -9.03171 | 2.03E-12 | *** |
| Hours12 - Hours48 | -0.10934 | 0.00418 | 5964 | -26.17564 | 1.96E-12 | *** |
| Hours18 - Hours24 | -0.06732 | 0.00418 | 5964 | -16.11573 | 1.96E-12 | *** |
| Hours18 - Hours48 | -0.13893 | 0.00418 | 5964 | -33.25966 | 1.96E-12 | *** |
| Hours24 - Hours48 | -0.07161 | 0.00418 | 5964 | -17.14393 | 1.96E-12 | *** |
| **Figure 2C** |  |  |  |  |  |  |
| (Hours-1) - Hours0 | 0.17756 | 0.00390 | 6120 | 45.54564 | 1.51E-12 | *** |
| (Hours-1) - Hours6 | 0.23449 | 0.00390 | 6120 | 60.14963 | 1.51E-12 | *** |
| (Hours-1) - Hours12 | 0.25049 | 0.00390 | 6120 | 64.25206 | 1.51E-12 | *** |
| (Hours-1) - Hours18 | 0.25856 | 0.00390 | 6120 | 66.32328 | 1.51E-12 | *** |
| (Hours-1) - Hours24 | 0.23305 | 0.00390 | 6120 | 59.78009 | 1.51E-12 | *** |
| (Hours-1) - Hours48 | 0.11968 | 0.00390 | 6120 | 30.69904 | 1.51E-12 | *** |
| Hours0 - Hours6 | 0.05693 | 0.00390 | 6120 | 14.60399 | 1.51E-12 | *** |
| Hours0 - Hours12 | 0.07293 | 0.00390 | 6120 | 18.70642 | 1.51E-12 | *** |
| Hours0 - Hours18 | 0.08100 | 0.00390 | 6120 | 20.77763 | 1.51E-12 | *** |
| Hours0 - Hours24 | 0.05549 | 0.00390 | 6120 | 14.23444 | 1.51E-12 | *** |
| Hours0 - Hours48 | -0.05788 | 0.00390 | 6120 | -14.84660 | 1.51E-12 | *** |
| Hours6 - Hours12 | 0.01599 | 0.00390 | 6120 | 4.10243 | 8.17E-04 | *** |
| Hours6 - Hours18 | 0.02407 | 0.00390 | 6120 | 6.17365 | 1.49E-08 | *** |
| Hours6 - Hours24 | -0.00144 | 0.00390 | 6120 | -0.36954 | 1.00E+00 | ns |
| Hours6 - Hours48 | -0.11481 | 0.00390 | 6120 | -29.45059 | 1.51E-12 | *** |
| Hours12 - Hours18 | 0.00807 | 0.00390 | 6120 | 2.07122 | 3.70E-01 | ns |
| Hours12 - Hours24 | -0.01743 | 0.00390 | 6120 | -4.47197 | 1.60E-04 | *** |
| Hours12 - Hours48 | -0.13081 | 0.00390 | 6120 | -33.55302 | 1.51E-12 | *** |
| Hours18 - Hours24 | -0.02551 | 0.00390 | 6120 | -6.54319 | 1.37E-09 | *** |
| Hours18 - Hours48 | -0.13888 | 0.00390 | 6120 | -35.62424 | 1.51E-12 | *** |
| Hours24 - Hours48 | -0.11337 | 0.00390 | 6120 | -29.08105 | 1.51E-12 | *** |
| **Figure 2D** |  |  |  |  |  |  |
| (Hours-1) - Hours0 | -0.00761 | 0.00452 | 6138 | -1.68329 | 6.27E-01 | ns |
| (Hours-1) - Hours6 | 0.06653 | 0.00452 | 6138 | 14.71461 | 0.00E+00 | *** |
| (Hours-1) - Hours12 | 0.09099 | 0.00452 | 6138 | 20.12702 | 0.00E+00 | *** |
| (Hours-1) - Hours18 | 0.08638 | 0.00452 | 6138 | 19.10580 | 0.00E+00 | *** |
| (Hours-1) - Hours24 | 0.04820 | 0.00452 | 6138 | 10.66052 | 0.00E+00 | *** |
| (Hours-1) - Hours48 | -0.06089 | 0.00452 | 6138 | -13.46775 | 0.00E+00 | *** |
| Hours0 - Hours6 | 0.07414 | 0.00452 | 6138 | 16.39789 | 0.00E+00 | *** |
| Hours0 - Hours12 | 0.09860 | 0.00452 | 6138 | 21.81030 | 0.00E+00 | *** |
| Hours0 - Hours18 | 0.09399 | 0.00452 | 6138 | 20.78909 | 0.00E+00 | *** |
| Hours0 - Hours24 | 0.05581 | 0.00452 | 6138 | 12.34381 | 0.00E+00 | *** |
| Hours0 - Hours48 | -0.05328 | 0.00452 | 6138 | -11.78446 | 0.00E+00 | *** |
| Hours6 - Hours12 | 0.02447 | 0.00452 | 6138 | 5.41241 | 1.35E-06 | *** |
| Hours6 - Hours18 | 0.01985 | 0.00452 | 6138 | 4.39119 | 2.31E-04 | *** |
| Hours6 - Hours24 | -0.01833 | 0.00452 | 6138 | -4.05408 | 1.00E-03 | ** |
| Hours6 - Hours48 | -0.12741 | 0.00452 | 6138 | -28.18236 | 0.00E+00 | *** |
| Hours12 - Hours18 | -0.00462 | 0.00452 | 6138 | -1.02122 | 9.49E-01 | ns |
| Hours12 - Hours24 | -0.04280 | 0.00452 | 6138 | -9.46649 | 0.00E+00 | *** |
| Hours12 - Hours48 | -0.15188 | 0.00452 | 6138 | -33.59477 | 0.00E+00 | *** |
| Hours18 - Hours24 | -0.03818 | 0.00452 | 6138 | -8.44528 | 0.00E+00 | *** |
| Hours18 - Hours48 | -0.14727 | 0.00452 | 6138 | -32.57355 | 0.00E+00 | *** |
| Hours24 - Hours48 | -0.10908 | 0.00452 | 6138 | -24.12827 | 0.00E+00 | *** |

**Supplementary Table S3:** Statistical results from linear mixed-effects model (LMM) for Figure 3B

| **Figure 3B** |  |  |  |  |  |  |
| --- | --- | --- | --- | --- | --- | --- |
| **Contrast** | **Estimate** | **SE** | **df** | **t.ratio** | **p.value** | **Significance** |
| (Hours-1.0) - Hours0.0 | 0.05152 | 0.00111 | 13299 | 46.58334 | 4.8756E-10 | *** |
| (Hours-1.0) - Hours0.5 | 0.05461 | 0.00111 | 13299 | 49.37247 | 4.8756E-10 | *** |
| (Hours-1.0) - Hours1.0 | 0.06375 | 0.00111 | 13299 | 57.63318 | 4.8756E-10 | *** |
| (Hours-1.0) - Hours1.5 | 0.07295 | 0.00111 | 13299 | 65.95395 | 4.8756E-10 | *** |
| (Hours-1.0) - Hours2.0 | 0.09390 | 0.00111 | 13299 | 84.89763 | 4.8756E-10 | *** |
| (Hours-1.0) - Hours2.25 | 0.10132 | 0.00111 | 13299 | 91.60445 | 4.8756E-10 | *** |
| (Hours-1.0) - Hours2.5 | 0.10598 | 0.00111 | 13299 | 95.81940 | 4.8756E-10 | *** |
| (Hours-1.0) - Hours2.75 | 0.11094 | 0.00111 | 13299 | 100.29948 | 4.8756E-10 | *** |
| (Hours-1.0) - Hours3.0 | 0.11506 | 0.00111 | 13299 | 104.02391 | 4.8756E-10 | *** |
| (Hours-1.0) - Hours3.25 | 0.11506 | 0.00111 | 13299 | 104.02391 | 4.8756E-10 | *** |
| (Hours-1.0) - Hours3.5 | 0.11790 | 0.00111 | 13299 | 106.59237 | 4.8756E-10 | *** |
| (Hours-1.0) - Hours3.75 | 0.12126 | 0.00111 | 13299 | 109.62739 | 4.8756E-10 | *** |
| (Hours-1.0) - Hours4.0 | 0.12368 | 0.00111 | 13299 | 111.81580 | 4.8756E-10 | *** |
| Hours0.0 - Hours0.5 | 0.00309 | 0.00111 | 13299 | 2.78913 | 2.2989E-01 | ns |
| Hours0.0 - Hours1.0 | 0.01222 | 0.00111 | 13299 | 11.04984 | 4.8764E-10 | *** |
| Hours0.0 - Hours1.5 | 0.02143 | 0.00111 | 13299 | 19.37060 | 4.8756E-10 | *** |
| Hours0.0 - Hours2.0 | 0.04238 | 0.00111 | 13299 | 38.31429 | 4.8756E-10 | *** |
| Hours0.0 - Hours2.25 | 0.04980 | 0.00111 | 13299 | 45.02111 | 4.8756E-10 | *** |
| Hours0.0 - Hours2.5 | 0.05446 | 0.00111 | 13299 | 49.23606 | 4.8756E-10 | *** |
| Hours0.0 - Hours2.75 | 0.05941 | 0.00111 | 13299 | 53.71614 | 4.8756E-10 | *** |
| Hours0.0 - Hours3.0 | 0.06353 | 0.00111 | 13299 | 57.44057 | 4.8756E-10 | *** |
| Hours0.0 - Hours3.25 | 0.06353 | 0.00111 | 13299 | 57.44057 | 4.8756E-10 | *** |
| Hours0.0 - Hours3.5 | 0.06637 | 0.00111 | 13299 | 60.00903 | 4.8756E-10 | *** |
| Hours0.0 - Hours3.75 | 0.06973 | 0.00111 | 13299 | 63.04405 | 4.8756E-10 | *** |
| Hours0.0 - Hours4.0 | 0.07215 | 0.00111 | 13299 | 65.23246 | 4.8756E-10 | *** |
| Hours0.5 - Hours1.0 | 0.00914 | 0.00111 | 13299 | 8.26071 | 4.8773E-10 | *** |
| Hours0.5 - Hours1.5 | 0.01834 | 0.00111 | 13299 | 16.58147 | 4.8756E-10 | *** |
| Hours0.5 - Hours2.0 | 0.03929 | 0.00111 | 13299 | 35.52516 | 4.8756E-10 | *** |
| Hours0.5 - Hours2.25 | 0.04671 | 0.00111 | 13299 | 42.23198 | 4.8756E-10 | *** |
| Hours0.5 - Hours2.5 | 0.05137 | 0.00111 | 13299 | 46.44693 | 4.8756E-10 | *** |
| Hours0.5 - Hours2.75 | 0.05633 | 0.00111 | 13299 | 50.92701 | 4.8756E-10 | *** |
| Hours0.5 - Hours3.0 | 0.06045 | 0.00111 | 13299 | 54.65144 | 4.8756E-10 | *** |
| Hours0.5 - Hours3.25 | 0.06045 | 0.00111 | 13299 | 54.65144 | 4.8756E-10 | *** |
| Hours0.5 - Hours3.5 | 0.06329 | 0.00111 | 13299 | 57.21990 | 4.8756E-10 | *** |
| Hours0.5 - Hours3.75 | 0.06665 | 0.00111 | 13299 | 60.25492 | 4.8756E-10 | *** |
| Hours0.5 - Hours4.0 | 0.06907 | 0.00111 | 13299 | 62.44333 | 4.8756E-10 | *** |
| Hours1.0 - Hours1.5 | 0.00920 | 0.00111 | 13299 | 8.32076 | 4.8772E-10 | *** |
| Hours1.0 - Hours2.0 | 0.03016 | 0.00111 | 13299 | 27.26445 | 4.8756E-10 | *** |
| Hours1.0 - Hours2.25 | 0.03757 | 0.00111 | 13299 | 33.97127 | 4.8756E-10 | *** |
| Hours1.0 - Hours2.5 | 0.04224 | 0.00111 | 13299 | 38.18622 | 4.8756E-10 | *** |
| Hours1.0 - Hours2.75 | 0.04719 | 0.00111 | 13299 | 42.66630 | 4.8756E-10 | *** |
| Hours1.0 - Hours3.0 | 0.05131 | 0.00111 | 13299 | 46.39073 | 4.8756E-10 | *** |
| Hours1.0 - Hours3.25 | 0.05131 | 0.00111 | 13299 | 46.39073 | 4.8756E-10 | *** |
| Hours1.0 - Hours3.5 | 0.05415 | 0.00111 | 13299 | 48.95919 | 4.8756E-10 | *** |
| Hours1.0 - Hours3.75 | 0.05751 | 0.00111 | 13299 | 51.99421 | 4.8756E-10 | *** |
| Hours1.0 - Hours4.0 | 0.05993 | 0.00111 | 13299 | 54.18262 | 4.8756E-10 | *** |
| Hours1.5 - Hours2.0 | 0.02095 | 0.00111 | 13299 | 18.94368 | 4.8756E-10 | *** |
| Hours1.5 - Hours2.25 | 0.02837 | 0.00111 | 13299 | 25.65051 | 4.8756E-10 | *** |
| Hours1.5 - Hours2.5 | 0.03303 | 0.00111 | 13299 | 29.86546 | 4.8756E-10 | *** |
| Hours1.5 - Hours2.75 | 0.03799 | 0.00111 | 13299 | 34.34554 | 4.8756E-10 | *** |
| Hours1.5 - Hours3.0 | 0.04211 | 0.00111 | 13299 | 38.06996 | 4.8756E-10 | *** |
| Hours1.5 - Hours3.25 | 0.04211 | 0.00111 | 13299 | 38.06996 | 4.8756E-10 | *** |
| Hours1.5 - Hours3.5 | 0.04495 | 0.00111 | 13299 | 40.63843 | 4.8756E-10 | *** |
| Hours1.5 - Hours3.75 | 0.04831 | 0.00111 | 13299 | 43.67345 | 4.8756E-10 | *** |
| Hours1.5 - Hours4.0 | 0.05073 | 0.00111 | 13299 | 45.86186 | 4.8756E-10 | *** |
| Hours2.0 - Hours2.25 | 0.00742 | 0.00111 | 13299 | 6.70682 | 2.3690E-09 | *** |
| Hours2.0 - Hours2.5 | 0.01208 | 0.00111 | 13299 | 10.92177 | 4.8768E-10 | *** |
| Hours2.0 - Hours2.75 | 0.01704 | 0.00111 | 13299 | 15.40185 | 4.8756E-10 | *** |
| Hours2.0 - Hours3.0 | 0.02116 | 0.00111 | 13299 | 19.12628 | 4.8756E-10 | *** |
| Hours2.0 - Hours3.25 | 0.02116 | 0.00111 | 13299 | 19.12628 | 4.8756E-10 | *** |
| Hours2.0 - Hours3.5 | 0.02400 | 0.00111 | 13299 | 21.69474 | 4.8756E-10 | *** |
| Hours2.0 - Hours3.75 | 0.02735 | 0.00111 | 13299 | 24.72976 | 4.8756E-10 | *** |
| Hours2.0 - Hours4.0 | 0.02977 | 0.00111 | 13299 | 26.91817 | 4.8756E-10 | *** |
| Hours2.25 - Hours2.5 | 0.00466 | 0.00111 | 13299 | 4.21495 | 2.0429E-03 | ** |
| Hours2.25 - Hours2.75 | 0.00962 | 0.00111 | 13299 | 8.69503 | 4.8767E-10 | *** |
| Hours2.25 - Hours3.0 | 0.01374 | 0.00111 | 13299 | 12.41945 | 4.8756E-10 | *** |
| Hours2.25 - Hours3.25 | 0.01374 | 0.00111 | 13299 | 12.41945 | 4.8756E-10 | *** |
| Hours2.25 - Hours3.5 | 0.01658 | 0.00111 | 13299 | 14.98792 | 4.8756E-10 | *** |
| Hours2.25 - Hours3.75 | 0.01993 | 0.00111 | 13299 | 18.02294 | 4.8756E-10 | *** |
| Hours2.25 - Hours4.0 | 0.02236 | 0.00111 | 13299 | 20.21135 | 4.8756E-10 | *** |
| Hours2.5 - Hours2.75 | 0.00496 | 0.00111 | 13299 | 4.48008 | 6.3493E-04 | *** |
| Hours2.5 - Hours3.0 | 0.00907 | 0.00111 | 13299 | 8.20450 | 4.8774E-10 | *** |
| Hours2.5 - Hours3.25 | 0.00907 | 0.00111 | 13299 | 8.20450 | 4.8774E-10 | *** |
| Hours2.5 - Hours3.5 | 0.01192 | 0.00111 | 13299 | 10.77297 | 4.8769E-10 | *** |
| Hours2.5 - Hours3.75 | 0.01527 | 0.00111 | 13299 | 13.80799 | 4.8756E-10 | *** |
| Hours2.5 - Hours4.0 | 0.01769 | 0.00111 | 13299 | 15.99640 | 4.8756E-10 | *** |
| Hours2.75 - Hours3.0 | 0.00412 | 0.00111 | 13299 | 3.72443 | 1.4226E-02 | * |
| Hours2.75 - Hours3.25 | 0.00412 | 0.00111 | 13299 | 3.72443 | 1.4226E-02 | * |
| Hours2.75 - Hours3.5 | 0.00696 | 0.00111 | 13299 | 6.29289 | 2.9661E-08 | *** |
| Hours2.75 - Hours3.75 | 0.01032 | 0.00111 | 13299 | 9.32791 | 4.8770E-10 | *** |
| Hours2.75 - Hours4.0 | 0.01274 | 0.00111 | 13299 | 11.51632 | 4.8756E-10 | *** |
| Hours3.0 - Hours3.25 | 0.00000 | 0.00111 | 13299 | 0.00000 | 1.0000E+00 | ns |
| Hours3.0 - Hours3.5 | 0.00284 | 0.00111 | 13299 | 2.56847 | 3.6095E-01 | ns |
| Hours3.0 - Hours3.75 | 0.00620 | 0.00111 | 13299 | 5.60349 | 1.9301E-06 | *** |
| Hours3.0 - Hours4.0 | 0.00862 | 0.00111 | 13299 | 7.79190 | 4.8830E-10 | *** |
| Hours3.25 - Hours3.5 | 0.00284 | 0.00111 | 13299 | 2.56847 | 3.6095E-01 | ns |
| Hours3.25 - Hours3.75 | 0.00620 | 0.00111 | 13299 | 5.60349 | 1.9301E-06 | *** |
| Hours3.25 - Hours4.0 | 0.00862 | 0.00111 | 13299 | 7.79190 | 4.8830E-10 | *** |
| Hours3.5 - Hours3.75 | 0.00336 | 0.00111 | 13299 | 3.03502 | 1.2601E-01 | ns |
| Hours3.5 - Hours4.0 | 0.00578 | 0.00111 | 13299 | 5.22343 | 1.5883E-05 | *** |
| Hours3.75 - Hours4.0 | 0.00242 | 0.00111 | 13299 | 2.18841 | 6.3817E-01 | ns |

**Supplementary Table S4:** Statistical results from linear mixed-effects model (LMM) for Figure 5B

| **Figure 5B** |  |  |  |  |  |  |  |
| --- | --- | --- | --- | --- | --- | --- | --- |
| **Intra-group difference** | | | | | | | |
| **Contrast** | **Treatment** | **Estimate** | **SE** | **df** | **t.ratio** | **p.value** | **Significance** |
| Hours0 - Hours4 | Ctrl | 0.01335 | 0.00491 | 5115 | 2.71985 | 1.7994E-02 | * |
| Hours0 - Hours7 | Ctrl | 0.02973 | 0.00491 | 5115 | 6.05793 | 4.4277E-09 | *** |
| Hours4 - Hours7 | Ctrl | 0.01638 | 0.00491 | 5115 | 3.33808 | 2.4477E-03 | ** |
| Hours0 - Hours4 | OD | -0.19130 | 0.00491 | 5115 | -38.98440 | 0.0000E+00 | *** |
| Hours0 - Hours7 | OD | -0.35595 | 0.00491 | 5115 | -72.53899 | 0.0000E+00 | *** |
| Hours4 - Hours7 | OD | -0.16465 | 0.00491 | 5115 | -33.55460 | 0.0000E+00 | *** |
| **Inter-group difference** | | | | | |  |  |
| **contrast** | **Hours** | **estimate** | **SE** | **df** | **t.ratio** | **p.value** | **Significance** |
| Ctrl - OD | 0 | -0.00529 | 0.00491 | 5115 | -1.07726 | 2.8141E-01 | ns |
| Ctrl - OD | 4 | -0.20993 | 0.00491 | 5115 | -42.78151 | 0.0000E+00 | *** |
| Ctrl - OD | 7 | -0.39096 | 0.00491 | 5115 | -79.67418 | 0.0000E+00 | *** |

**Supplementary Table S5:** Statistical results from linear mixed-effects model (LMM) for Figure S2C

| **Figure S2C** |  |  |  |  |  |  |
| --- | --- | --- | --- | --- | --- | --- |
| **Contrast** | **Estimate** | **SE** | **df** | **t.ratio** | **p.value** | **Significance** |
| (Hours-1.0) - Hours0.0 | 0.07020 | 0.00320 | 22484 | 21.94736 | 0.000E+00 | *** |
| (Hours-1.0) - Hours0.25 | 0.07158 | 0.00320 | 22484 | 22.37841 | 0.000E+00 | *** |
| (Hours-1.0) - Hours0.5 | 0.07954 | 0.00320 | 22484 | 24.86790 | 0.000E+00 | *** |
| (Hours-1.0) - Hours0.75 | 0.08748 | 0.00320 | 22484 | 27.35064 | 0.000E+00 | *** |
| (Hours-1.0) - Hours1.0 | 0.09223 | 0.00320 | 22484 | 28.83491 | 0.000E+00 | *** |
| (Hours-1.0) - Hours1.25 | 0.09539 | 0.00320 | 22484 | 29.82375 | 0.000E+00 | *** |
| (Hours-1.0) - Hours1.5 | 0.09826 | 0.00320 | 22484 | 30.71869 | 0.000E+00 | *** |
| (Hours-1.0) - Hours1.75 | 0.09900 | 0.00320 | 22484 | 30.95122 | 0.000E+00 | *** |
| (Hours-1.0) - Hours2.0 | 0.09919 | 0.00320 | 22484 | 31.01059 | 0.000E+00 | *** |
| (Hours-1.0) - Hours2.25 | 0.10117 | 0.00320 | 22484 | 31.62908 | 0.000E+00 | *** |
| (Hours-1.0) - Hours2.5 | 0.10167 | 0.00320 | 22484 | 31.78668 | 0.000E+00 | *** |
| (Hours-1.0) - Hours3.0 | 0.00169 | 0.00320 | 22484 | 0.52881 | 1.000E+00 | ns |
| (Hours-1.0) - Hours3.25 | -0.01553 | 0.00320 | 22484 | -4.85427 | 2.877E-04 | *** |
| (Hours-1.0) - Hours3.5 | -0.02533 | 0.00320 | 22484 | -7.91814 | 0.000E+00 | *** |
| (Hours-1.0) - Hours3.75 | -0.03203 | 0.00320 | 22484 | -10.01329 | 0.000E+00 | *** |
| (Hours-1.0) - Hours4.0 | -0.03805 | 0.00320 | 22484 | -11.89699 | 0.000E+00 | *** |
| (Hours-1.0) - Hours4.25 | -0.04194 | 0.00320 | 22484 | -13.11300 | 0.000E+00 | *** |
| (Hours-1.0) - Hours5.0 | -0.05962 | 0.00320 | 22484 | -18.63850 | 0.000E+00 | *** |
| (Hours-1.0) - Hours6.25 | -0.06407 | 0.00320 | 22484 | -20.03173 | 0.000E+00 | *** |
| (Hours-1.0) - Hours6.5 | -0.06418 | 0.00320 | 22484 | -20.06565 | 0.000E+00 | *** |
| (Hours-1.0) - Hours6.75 | -0.06440 | 0.00320 | 22484 | -20.13276 | 0.000E+00 | *** |
| (Hours-1.0) - Hours8.0 | -0.10724 | 0.00320 | 22484 | -33.52793 | 0.000E+00 | *** |
| Hours0.0 - Hours0.25 | 0.00138 | 0.00320 | 22484 | 0.43106 | 1.000E+00 | ns |
| Hours0.0 - Hours0.5 | 0.00934 | 0.00320 | 22484 | 2.92054 | 3.302E-01 | ns |
| Hours0.0 - Hours0.75 | 0.01728 | 0.00320 | 22484 | 5.40328 | 1.608E-05 | *** |
| Hours0.0 - Hours1.0 | 0.02203 | 0.00320 | 22484 | 6.88755 | 0.000E+00 | *** |
| Hours0.0 - Hours1.25 | 0.02519 | 0.00320 | 22484 | 7.87639 | 0.000E+00 | *** |
| Hours0.0 - Hours1.5 | 0.02806 | 0.00320 | 22484 | 8.77133 | 0.000E+00 | *** |
| Hours0.0 - Hours1.75 | 0.02880 | 0.00320 | 22484 | 9.00386 | 0.000E+00 | *** |
| Hours0.0 - Hours2.0 | 0.02899 | 0.00320 | 22484 | 9.06324 | 0.000E+00 | *** |
| Hours0.0 - Hours2.25 | 0.03097 | 0.00320 | 22484 | 9.68172 | 0.000E+00 | *** |
| Hours0.0 - Hours2.5 | 0.03147 | 0.00320 | 22484 | 9.83932 | 0.000E+00 | *** |
| Hours0.0 - Hours3.0 | -0.06851 | 0.00320 | 22484 | -21.41854 | 0.000E+00 | *** |
| Hours0.0 - Hours3.25 | -0.08573 | 0.00320 | 22484 | -26.80162 | 0.000E+00 | *** |
| Hours0.0 - Hours3.5 | -0.09553 | 0.00320 | 22484 | -29.86549 | 0.000E+00 | *** |
| Hours0.0 - Hours3.75 | -0.10223 | 0.00320 | 22484 | -31.96064 | 0.000E+00 | *** |
| Hours0.0 - Hours4.0 | -0.10825 | 0.00320 | 22484 | -33.84435 | 0.000E+00 | *** |
| Hours0.0 - Hours4.25 | -0.11214 | 0.00320 | 22484 | -35.06036 | 0.000E+00 | *** |
| Hours0.0 - Hours5.0 | -0.12982 | 0.00320 | 22484 | -40.58586 | 0.000E+00 | *** |
| Hours0.0 - Hours6.25 | -0.13427 | 0.00320 | 22484 | -41.97909 | 0.000E+00 | *** |
| Hours0.0 - Hours6.5 | -0.13438 | 0.00320 | 22484 | -42.01301 | 0.000E+00 | *** |
| Hours0.0 - Hours6.75 | -0.13460 | 0.00320 | 22484 | -42.08012 | 0.000E+00 | *** |
| Hours0.0 - Hours8.0 | -0.17744 | 0.00320 | 22484 | -55.47529 | 0.000E+00 | *** |
| Hours0.25 - Hours0.5 | 0.00796 | 0.00320 | 22484 | 2.48948 | 6.630E-01 | ns |
| Hours0.25 - Hours0.75 | 0.01590 | 0.00320 | 22484 | 4.97223 | 1.595E-04 | *** |
| Hours0.25 - Hours1.0 | 0.02065 | 0.00320 | 22484 | 6.45650 | 0.000E+00 | *** |
| Hours0.25 - Hours1.25 | 0.02381 | 0.00320 | 22484 | 7.44533 | 0.000E+00 | *** |
| Hours0.25 - Hours1.5 | 0.02668 | 0.00320 | 22484 | 8.34027 | 0.000E+00 | *** |
| Hours0.25 - Hours1.75 | 0.02742 | 0.00320 | 22484 | 8.57280 | 0.000E+00 | *** |
| Hours0.25 - Hours2.0 | 0.02761 | 0.00320 | 22484 | 8.63218 | 0.000E+00 | *** |
| Hours0.25 - Hours2.25 | 0.02959 | 0.00320 | 22484 | 9.25067 | 0.000E+00 | *** |
| Hours0.25 - Hours2.5 | 0.03009 | 0.00320 | 22484 | 9.40827 | 0.000E+00 | *** |
| Hours0.25 - Hours3.0 | -0.06989 | 0.00320 | 22484 | -21.84960 | 0.000E+00 | *** |
| Hours0.25 - Hours3.25 | -0.08711 | 0.00320 | 22484 | -27.23268 | 0.000E+00 | *** |
| Hours0.25 - Hours3.5 | -0.09691 | 0.00320 | 22484 | -30.29655 | 0.000E+00 | *** |
| Hours0.25 - Hours3.75 | -0.10361 | 0.00320 | 22484 | -32.39170 | 0.000E+00 | *** |
| Hours0.25 - Hours4.0 | -0.10963 | 0.00320 | 22484 | -34.27541 | 0.000E+00 | *** |
| Hours0.25 - Hours4.25 | -0.11352 | 0.00320 | 22484 | -35.49142 | 0.000E+00 | *** |
| Hours0.25 - Hours5.0 | -0.13120 | 0.00320 | 22484 | -41.01692 | 0.000E+00 | *** |
| Hours0.25 - Hours6.25 | -0.13565 | 0.00320 | 22484 | -42.41015 | 0.000E+00 | *** |
| Hours0.25 - Hours6.5 | -0.13576 | 0.00320 | 22484 | -42.44406 | 0.000E+00 | *** |
| Hours0.25 - Hours6.75 | -0.13598 | 0.00320 | 22484 | -42.51118 | 0.000E+00 | *** |
| Hours0.25 - Hours8.0 | -0.17882 | 0.00320 | 22484 | -55.90634 | 0.000E+00 | *** |
| Hours0.5 - Hours0.75 | 0.00794 | 0.00320 | 22484 | 2.48274 | 6.682E-01 | ns |
| Hours0.5 - Hours1.0 | 0.01269 | 0.00320 | 22484 | 3.96701 | 1.426E-02 | * |
| Hours0.5 - Hours1.25 | 0.01585 | 0.00320 | 22484 | 4.95585 | 1.733E-04 | *** |
| Hours0.5 - Hours1.5 | 0.01871 | 0.00320 | 22484 | 5.85079 | 1.014E-06 | *** |
| Hours0.5 - Hours1.75 | 0.01946 | 0.00320 | 22484 | 6.08332 | 7.308E-08 | *** |
| Hours0.5 - Hours2.0 | 0.01965 | 0.00320 | 22484 | 6.14269 | 0.000E+00 | *** |
| Hours0.5 - Hours2.25 | 0.02163 | 0.00320 | 22484 | 6.76118 | 0.000E+00 | *** |
| Hours0.5 - Hours2.5 | 0.02213 | 0.00320 | 22484 | 6.91878 | 0.000E+00 | *** |
| Hours0.5 - Hours3.0 | -0.07785 | 0.00320 | 22484 | -24.33908 | 0.000E+00 | *** |
| Hours0.5 - Hours3.25 | -0.09507 | 0.00320 | 22484 | -29.72217 | 0.000E+00 | *** |
| Hours0.5 - Hours3.5 | -0.10487 | 0.00320 | 22484 | -32.78603 | 0.000E+00 | *** |
| Hours0.5 - Hours3.75 | -0.11157 | 0.00320 | 22484 | -34.88119 | 0.000E+00 | *** |
| Hours0.5 - Hours4.0 | -0.11760 | 0.00320 | 22484 | -36.76489 | 0.000E+00 | *** |
| Hours0.5 - Hours4.25 | -0.12149 | 0.00320 | 22484 | -37.98090 | 0.000E+00 | *** |
| Hours0.5 - Hours5.0 | -0.13916 | 0.00320 | 22484 | -43.50640 | 0.000E+00 | *** |
| Hours0.5 - Hours6.25 | -0.14362 | 0.00320 | 22484 | -44.89963 | 0.000E+00 | *** |
| Hours0.5 - Hours6.5 | -0.14372 | 0.00320 | 22484 | -44.93355 | 0.000E+00 | *** |
| Hours0.5 - Hours6.75 | -0.14394 | 0.00320 | 22484 | -45.00066 | 0.000E+00 | *** |
| Hours0.5 - Hours8.0 | -0.18678 | 0.00320 | 22484 | -58.39583 | 0.000E+00 | *** |
| Hours0.75 - Hours1.0 | 0.00475 | 0.00320 | 22484 | 1.48427 | 9.984E-01 | ns |
| Hours0.75 - Hours1.25 | 0.00791 | 0.00320 | 22484 | 2.47311 | 6.756E-01 | ns |
| Hours0.75 - Hours1.5 | 0.01077 | 0.00320 | 22484 | 3.36805 | 1.081E-01 | ns |
| Hours0.75 - Hours1.75 | 0.01152 | 0.00320 | 22484 | 3.60058 | 5.263E-02 | ns |
| Hours0.75 - Hours2.0 | 0.01171 | 0.00320 | 22484 | 3.65995 | 4.318E-02 | * |
| Hours0.75 - Hours2.25 | 0.01369 | 0.00320 | 22484 | 4.27844 | 4.053E-03 | ** |
| Hours0.75 - Hours2.5 | 0.01419 | 0.00320 | 22484 | 4.43604 | 2.045E-03 | ** |
| Hours0.75 - Hours3.0 | -0.08579 | 0.00320 | 22484 | -26.82183 | 0.000E+00 | *** |
| Hours0.75 - Hours3.25 | -0.10301 | 0.00320 | 22484 | -32.20491 | 0.000E+00 | *** |
| Hours0.75 - Hours3.5 | -0.11281 | 0.00320 | 22484 | -35.26878 | 0.000E+00 | *** |
| Hours0.75 - Hours3.75 | -0.11951 | 0.00320 | 22484 | -37.36393 | 0.000E+00 | *** |
| Hours0.75 - Hours4.0 | -0.12554 | 0.00320 | 22484 | -39.24763 | 0.000E+00 | *** |
| Hours0.75 - Hours4.25 | -0.12943 | 0.00320 | 22484 | -40.46364 | 0.000E+00 | *** |
| Hours0.75 - Hours5.0 | -0.14710 | 0.00320 | 22484 | -45.98914 | 0.000E+00 | *** |
| Hours0.75 - Hours6.25 | -0.15156 | 0.00320 | 22484 | -47.38237 | 0.000E+00 | *** |
| Hours0.75 - Hours6.5 | -0.15167 | 0.00320 | 22484 | -47.41629 | 0.000E+00 | *** |
| Hours0.75 - Hours6.75 | -0.15188 | 0.00320 | 22484 | -47.48340 | 0.000E+00 | *** |
| Hours0.75 - Hours8.0 | -0.19473 | 0.00320 | 22484 | -60.87857 | 0.000E+00 | *** |
| Hours1.0 - Hours1.25 | 0.00316 | 0.00320 | 22484 | 0.98884 | 1.000E+00 | ns |
| Hours1.0 - Hours1.5 | 0.00603 | 0.00320 | 22484 | 1.88378 | 9.667E-01 | ns |
| Hours1.0 - Hours1.75 | 0.00677 | 0.00320 | 22484 | 2.11631 | 8.959E-01 | ns |
| Hours1.0 - Hours2.0 | 0.00696 | 0.00320 | 22484 | 2.17568 | 8.684E-01 | ns |
| Hours1.0 - Hours2.25 | 0.00894 | 0.00320 | 22484 | 2.79417 | 4.216E-01 | ns |
| Hours1.0 - Hours2.5 | 0.00944 | 0.00320 | 22484 | 2.95177 | 3.093E-01 | ns |
| Hours1.0 - Hours3.0 | -0.09054 | 0.00320 | 22484 | -28.30610 | 0.000E+00 | *** |
| Hours1.0 - Hours3.25 | -0.10776 | 0.00320 | 22484 | -33.68918 | 0.000E+00 | *** |
| Hours1.0 - Hours3.5 | -0.11756 | 0.00320 | 22484 | -36.75305 | 0.000E+00 | *** |
| Hours1.0 - Hours3.75 | -0.12426 | 0.00320 | 22484 | -38.84820 | 0.000E+00 | *** |
| Hours1.0 - Hours4.0 | -0.13029 | 0.00320 | 22484 | -40.73190 | 0.000E+00 | *** |
| Hours1.0 - Hours4.25 | -0.13417 | 0.00320 | 22484 | -41.94791 | 0.000E+00 | *** |
| Hours1.0 - Hours5.0 | -0.15185 | 0.00320 | 22484 | -47.47341 | 0.000E+00 | *** |
| Hours1.0 - Hours6.25 | -0.15630 | 0.00320 | 22484 | -48.86664 | 0.000E+00 | *** |
| Hours1.0 - Hours6.5 | -0.15641 | 0.00320 | 22484 | -48.90056 | 0.000E+00 | *** |
| Hours1.0 - Hours6.75 | -0.15663 | 0.00320 | 22484 | -48.96767 | 0.000E+00 | *** |
| Hours1.0 - Hours8.0 | -0.19947 | 0.00320 | 22484 | -62.36284 | 0.000E+00 | *** |
| Hours1.25 - Hours1.5 | 0.00286 | 0.00320 | 22484 | 0.89494 | 1.000E+00 | ns |
| Hours1.25 - Hours1.75 | 0.00361 | 0.00320 | 22484 | 1.12747 | 1.000E+00 | ns |
| Hours1.25 - Hours2.0 | 0.00380 | 0.00320 | 22484 | 1.18685 | 1.000E+00 | ns |
| Hours1.25 - Hours2.25 | 0.00577 | 0.00320 | 22484 | 1.80534 | 9.793E-01 | ns |
| Hours1.25 - Hours2.5 | 0.00628 | 0.00320 | 22484 | 1.96293 | 9.488E-01 | ns |
| Hours1.25 - Hours3.0 | -0.09370 | 0.00320 | 22484 | -29.29493 | 0.000E+00 | *** |
| Hours1.25 - Hours3.25 | -0.11092 | 0.00320 | 22484 | -34.67801 | 0.000E+00 | *** |
| Hours1.25 - Hours3.5 | -0.12072 | 0.00320 | 22484 | -37.74188 | 0.000E+00 | *** |
| Hours1.25 - Hours3.75 | -0.12742 | 0.00320 | 22484 | -39.83703 | 0.000E+00 | *** |
| Hours1.25 - Hours4.0 | -0.13345 | 0.00320 | 22484 | -41.72074 | 0.000E+00 | *** |
| Hours1.25 - Hours4.25 | -0.13734 | 0.00320 | 22484 | -42.93675 | 0.000E+00 | *** |
| Hours1.25 - Hours5.0 | -0.15501 | 0.00320 | 22484 | -48.46225 | 0.000E+00 | *** |
| Hours1.25 - Hours6.25 | -0.15947 | 0.00320 | 22484 | -49.85548 | 0.000E+00 | *** |
| Hours1.25 - Hours6.5 | -0.15958 | 0.00320 | 22484 | -49.88940 | 0.000E+00 | *** |
| Hours1.25 - Hours6.75 | -0.15979 | 0.00320 | 22484 | -49.95651 | 0.000E+00 | *** |
| Hours1.25 - Hours8.0 | -0.20264 | 0.00320 | 22484 | -63.35168 | 0.000E+00 | *** |
| Hours1.5 - Hours1.75 | 0.00074 | 0.00320 | 22484 | 0.23253 | 1.000E+00 | ns |
| Hours1.5 - Hours2.0 | 0.00093 | 0.00320 | 22484 | 0.29191 | 1.000E+00 | ns |
| Hours1.5 - Hours2.25 | 0.00291 | 0.00320 | 22484 | 0.91040 | 1.000E+00 | ns |
| Hours1.5 - Hours2.5 | 0.00342 | 0.00320 | 22484 | 1.06800 | 1.000E+00 | ns |
| Hours1.5 - Hours3.0 | -0.09657 | 0.00320 | 22484 | -30.18987 | 0.000E+00 | *** |
| Hours1.5 - Hours3.25 | -0.11378 | 0.00320 | 22484 | -35.57295 | 0.000E+00 | *** |
| Hours1.5 - Hours3.5 | -0.12358 | 0.00320 | 22484 | -38.63682 | 0.000E+00 | *** |
| Hours1.5 - Hours3.75 | -0.13029 | 0.00320 | 22484 | -40.73197 | 0.000E+00 | *** |
| Hours1.5 - Hours4.0 | -0.13631 | 0.00320 | 22484 | -42.61568 | 0.000E+00 | *** |
| Hours1.5 - Hours4.25 | -0.14020 | 0.00320 | 22484 | -43.83169 | 0.000E+00 | *** |
| Hours1.5 - Hours5.0 | -0.15787 | 0.00320 | 22484 | -49.35719 | 0.000E+00 | *** |
| Hours1.5 - Hours6.25 | -0.16233 | 0.00320 | 22484 | -50.75042 | 0.000E+00 | *** |
| Hours1.5 - Hours6.5 | -0.16244 | 0.00320 | 22484 | -50.78434 | 0.000E+00 | *** |
| Hours1.5 - Hours6.75 | -0.16265 | 0.00320 | 22484 | -50.85145 | 0.000E+00 | *** |
| Hours1.5 - Hours8.0 | -0.20550 | 0.00320 | 22484 | -64.24661 | 0.000E+00 | *** |
| Hours1.75 - Hours2.0 | 0.00019 | 0.00320 | 22484 | 0.05938 | 1.000E+00 | ns |
| Hours1.75 - Hours2.25 | 0.00217 | 0.00320 | 22484 | 0.67787 | 1.000E+00 | ns |
| Hours1.75 - Hours2.5 | 0.00267 | 0.00320 | 22484 | 0.83546 | 1.000E+00 | ns |
| Hours1.75 - Hours3.0 | -0.09731 | 0.00320 | 22484 | -30.42240 | 0.000E+00 | *** |
| Hours1.75 - Hours3.25 | -0.11453 | 0.00320 | 22484 | -35.80548 | 0.000E+00 | *** |
| Hours1.75 - Hours3.5 | -0.12433 | 0.00320 | 22484 | -38.86935 | 0.000E+00 | *** |
| Hours1.75 - Hours3.75 | -0.13103 | 0.00320 | 22484 | -40.96450 | 0.000E+00 | *** |
| Hours1.75 - Hours4.0 | -0.13705 | 0.00320 | 22484 | -42.84821 | 0.000E+00 | *** |
| Hours1.75 - Hours4.25 | -0.14094 | 0.00320 | 22484 | -44.06422 | 0.000E+00 | *** |
| Hours1.75 - Hours5.0 | -0.15862 | 0.00320 | 22484 | -49.58972 | 0.000E+00 | *** |
| Hours1.75 - Hours6.25 | -0.16307 | 0.00320 | 22484 | -50.98295 | 0.000E+00 | *** |
| Hours1.75 - Hours6.5 | -0.16318 | 0.00320 | 22484 | -51.01687 | 0.000E+00 | *** |
| Hours1.75 - Hours6.75 | -0.16340 | 0.00320 | 22484 | -51.08398 | 0.000E+00 | *** |
| Hours1.75 - Hours8.0 | -0.20624 | 0.00320 | 22484 | -64.47915 | 0.000E+00 | *** |
| Hours2.0 - Hours2.25 | 0.00198 | 0.00320 | 22484 | 0.61849 | 1.000E+00 | ns |
| Hours2.0 - Hours2.5 | 0.00248 | 0.00320 | 22484 | 0.77609 | 1.000E+00 | ns |
| Hours2.0 - Hours3.0 | -0.09750 | 0.00320 | 22484 | -30.48178 | 0.000E+00 | *** |
| Hours2.0 - Hours3.25 | -0.11472 | 0.00320 | 22484 | -35.86486 | 0.000E+00 | *** |
| Hours2.0 - Hours3.5 | -0.12452 | 0.00320 | 22484 | -38.92873 | 0.000E+00 | *** |
| Hours2.0 - Hours3.75 | -0.13122 | 0.00320 | 22484 | -41.02388 | 0.000E+00 | *** |
| Hours2.0 - Hours4.0 | -0.13724 | 0.00320 | 22484 | -42.90759 | 0.000E+00 | *** |
| Hours2.0 - Hours4.25 | -0.14113 | 0.00320 | 22484 | -44.12360 | 0.000E+00 | *** |
| Hours2.0 - Hours5.0 | -0.15881 | 0.00320 | 22484 | -49.64910 | 0.000E+00 | *** |
| Hours2.0 - Hours6.25 | -0.16326 | 0.00320 | 22484 | -51.04233 | 0.000E+00 | *** |
| Hours2.0 - Hours6.5 | -0.16337 | 0.00320 | 22484 | -51.07624 | 0.000E+00 | *** |
| Hours2.0 - Hours6.75 | -0.16359 | 0.00320 | 22484 | -51.14336 | 0.000E+00 | *** |
| Hours2.0 - Hours8.0 | -0.20643 | 0.00320 | 22484 | -64.53852 | 0.000E+00 | *** |
| Hours2.25 - Hours2.5 | 0.00050 | 0.00320 | 22484 | 0.15760 | 1.000E+00 | ns |
| Hours2.25 - Hours3.0 | -0.09948 | 0.00320 | 22484 | -31.10027 | 0.000E+00 | *** |
| Hours2.25 - Hours3.25 | -0.11670 | 0.00320 | 22484 | -36.48335 | 0.000E+00 | *** |
| Hours2.25 - Hours3.5 | -0.12650 | 0.00320 | 22484 | -39.54722 | 0.000E+00 | *** |
| Hours2.25 - Hours3.75 | -0.13320 | 0.00320 | 22484 | -41.64237 | 0.000E+00 | *** |
| Hours2.25 - Hours4.0 | -0.13922 | 0.00320 | 22484 | -43.52607 | 0.000E+00 | *** |
| Hours2.25 - Hours4.25 | -0.14311 | 0.00320 | 22484 | -44.74208 | 0.000E+00 | *** |
| Hours2.25 - Hours5.0 | -0.16079 | 0.00320 | 22484 | -50.26759 | 0.000E+00 | *** |
| Hours2.25 - Hours6.25 | -0.16524 | 0.00320 | 22484 | -51.66082 | 0.000E+00 | *** |
| Hours2.25 - Hours6.5 | -0.16535 | 0.00320 | 22484 | -51.69473 | 0.000E+00 | *** |
| Hours2.25 - Hours6.75 | -0.16557 | 0.00320 | 22484 | -51.76185 | 0.000E+00 | *** |
| Hours2.25 - Hours8.0 | -0.20841 | 0.00320 | 22484 | -65.15701 | 0.000E+00 | *** |
| Hours2.5 - Hours3.0 | -0.09998 | 0.00320 | 22484 | -31.25787 | 0.000E+00 | *** |
| Hours2.5 - Hours3.25 | -0.11720 | 0.00320 | 22484 | -36.64095 | 0.000E+00 | *** |
| Hours2.5 - Hours3.5 | -0.12700 | 0.00320 | 22484 | -39.70482 | 0.000E+00 | *** |
| Hours2.5 - Hours3.75 | -0.13370 | 0.00320 | 22484 | -41.79997 | 0.000E+00 | *** |
| Hours2.5 - Hours4.0 | -0.13973 | 0.00320 | 22484 | -43.68367 | 0.000E+00 | *** |
| Hours2.5 - Hours4.25 | -0.14362 | 0.00320 | 22484 | -44.89968 | 0.000E+00 | *** |
| Hours2.5 - Hours5.0 | -0.16129 | 0.00320 | 22484 | -50.42519 | 0.000E+00 | *** |
| Hours2.5 - Hours6.25 | -0.16575 | 0.00320 | 22484 | -51.81841 | 0.000E+00 | *** |
| Hours2.5 - Hours6.5 | -0.16585 | 0.00320 | 22484 | -51.85233 | 0.000E+00 | *** |
| Hours2.5 - Hours6.75 | -0.16607 | 0.00320 | 22484 | -51.91945 | 0.000E+00 | *** |
| Hours2.5 - Hours8.0 | -0.20892 | 0.00320 | 22484 | -65.31461 | 0.000E+00 | *** |
| Hours3.0 - Hours3.25 | -0.01722 | 0.00320 | 22484 | -5.38308 | 1.800E-05 | *** |
| Hours3.0 - Hours3.5 | -0.02702 | 0.00320 | 22484 | -8.44695 | 0.000E+00 | *** |
| Hours3.0 - Hours3.75 | -0.03372 | 0.00320 | 22484 | -10.54210 | 0.000E+00 | *** |
| Hours3.0 - Hours4.0 | -0.03975 | 0.00320 | 22484 | -12.42581 | 0.000E+00 | *** |
| Hours3.0 - Hours4.25 | -0.04363 | 0.00320 | 22484 | -13.64182 | 0.000E+00 | *** |
| Hours3.0 - Hours5.0 | -0.06131 | 0.00320 | 22484 | -19.16732 | 0.000E+00 | *** |
| Hours3.0 - Hours6.25 | -0.06576 | 0.00320 | 22484 | -20.56055 | 0.000E+00 | *** |
| Hours3.0 - Hours6.5 | -0.06587 | 0.00320 | 22484 | -20.59446 | 0.000E+00 | *** |
| Hours3.0 - Hours6.75 | -0.06609 | 0.00320 | 22484 | -20.66158 | 0.000E+00 | *** |
| Hours3.0 - Hours8.0 | -0.10893 | 0.00320 | 22484 | -34.05674 | 0.000E+00 | *** |
| Hours3.25 - Hours3.5 | -0.00980 | 0.00320 | 22484 | -3.06387 | 2.407E-01 | ns |
| Hours3.25 - Hours3.75 | -0.01650 | 0.00320 | 22484 | -5.15902 | 6.066E-05 | *** |
| Hours3.25 - Hours4.0 | -0.02253 | 0.00320 | 22484 | -7.04273 | 0.000E+00 | *** |
| Hours3.25 - Hours4.25 | -0.02642 | 0.00320 | 22484 | -8.25874 | 0.000E+00 | *** |
| Hours3.25 - Hours5.0 | -0.04409 | 0.00320 | 22484 | -13.78424 | 0.000E+00 | *** |
| Hours3.25 - Hours6.25 | -0.04855 | 0.00320 | 22484 | -15.17747 | 0.000E+00 | *** |
| Hours3.25 - Hours6.5 | -0.04866 | 0.00320 | 22484 | -15.21138 | 0.000E+00 | *** |
| Hours3.25 - Hours6.75 | -0.04887 | 0.00320 | 22484 | -15.27850 | 0.000E+00 | *** |
| Hours3.25 - Hours8.0 | -0.09172 | 0.00320 | 22484 | -28.67366 | 0.000E+00 | *** |
| Hours3.5 - Hours3.75 | -0.00670 | 0.00320 | 22484 | -2.09515 | 9.048E-01 | ns |
| Hours3.5 - Hours4.0 | -0.01273 | 0.00320 | 22484 | -3.97886 | 1.363E-02 | * |
| Hours3.5 - Hours4.25 | -0.01662 | 0.00320 | 22484 | -5.19487 | 5.015E-05 | *** |
| Hours3.5 - Hours5.0 | -0.03429 | 0.00320 | 22484 | -10.72037 | 0.000E+00 | *** |
| Hours3.5 - Hours6.25 | -0.03875 | 0.00320 | 22484 | -12.11360 | 0.000E+00 | *** |
| Hours3.5 - Hours6.5 | -0.03886 | 0.00320 | 22484 | -12.14751 | 0.000E+00 | *** |
| Hours3.5 - Hours6.75 | -0.03907 | 0.00320 | 22484 | -12.21463 | 0.000E+00 | *** |
| Hours3.5 - Hours8.0 | -0.08192 | 0.00320 | 22484 | -25.60979 | 0.000E+00 | *** |
| Hours3.75 - Hours4.0 | -0.00603 | 0.00320 | 22484 | -1.88371 | 9.667E-01 | ns |
| Hours3.75 - Hours4.25 | -0.00991 | 0.00320 | 22484 | -3.09972 | 2.210E-01 | ns |
| Hours3.75 - Hours5.0 | -0.02759 | 0.00320 | 22484 | -8.62522 | 0.000E+00 | *** |
| Hours3.75 - Hours6.25 | -0.03205 | 0.00320 | 22484 | -10.01845 | 0.000E+00 | *** |
| Hours3.75 - Hours6.5 | -0.03215 | 0.00320 | 22484 | -10.05236 | 0.000E+00 | *** |
| Hours3.75 - Hours6.75 | -0.03237 | 0.00320 | 22484 | -10.11948 | 0.000E+00 | *** |
| Hours3.75 - Hours8.0 | -0.07521 | 0.00320 | 22484 | -23.51464 | 0.000E+00 | *** |
| Hours4.0 - Hours4.25 | -0.00389 | 0.00320 | 22484 | -1.21601 | 9.999E-01 | ns |
| Hours4.0 - Hours5.0 | -0.02156 | 0.00320 | 22484 | -6.74151 | 0.000E+00 | *** |
| Hours4.0 - Hours6.25 | -0.02602 | 0.00320 | 22484 | -8.13474 | 0.000E+00 | *** |
| Hours4.0 - Hours6.5 | -0.02613 | 0.00320 | 22484 | -8.16866 | 0.000E+00 | *** |
| Hours4.0 - Hours6.75 | -0.02634 | 0.00320 | 22484 | -8.23577 | 0.000E+00 | *** |
| Hours4.0 - Hours8.0 | -0.06919 | 0.00320 | 22484 | -21.63094 | 0.000E+00 | *** |
| Hours4.25 - Hours5.0 | -0.01767 | 0.00320 | 22484 | -5.52550 | 8.009E-06 | *** |
| Hours4.25 - Hours6.25 | -0.02213 | 0.00320 | 22484 | -6.91873 | 0.000E+00 | *** |
| Hours4.25 - Hours6.5 | -0.02224 | 0.00320 | 22484 | -6.95265 | 0.000E+00 | *** |
| Hours4.25 - Hours6.75 | -0.02245 | 0.00320 | 22484 | -7.01976 | 0.000E+00 | *** |
| Hours4.25 - Hours8.0 | -0.06530 | 0.00320 | 22484 | -20.41493 | 0.000E+00 | *** |
| Hours5.0 - Hours6.25 | -0.00446 | 0.00320 | 22484 | -1.39323 | 9.994E-01 | ns |
| Hours5.0 - Hours6.5 | -0.00456 | 0.00320 | 22484 | -1.42715 | 9.991E-01 | ns |
| Hours5.0 - Hours6.75 | -0.00478 | 0.00320 | 22484 | -1.49426 | 9.982E-01 | ns |
| Hours5.0 - Hours8.0 | -0.04763 | 0.00320 | 22484 | -14.88942 | 0.000E+00 | *** |
| Hours6.25 - Hours6.5 | -0.00011 | 0.00320 | 22484 | -0.03392 | 1.000E+00 | ns |
| Hours6.25 - Hours6.75 | -0.00032 | 0.00320 | 22484 | -0.10103 | 1.000E+00 | ns |
| Hours6.25 - Hours8.0 | -0.04317 | 0.00320 | 22484 | -13.49620 | 0.000E+00 | *** |
| Hours6.5 - Hours6.75 | -0.00021 | 0.00320 | 22484 | -0.06711 | 1.000E+00 | ns |
| Hours6.5 - Hours8.0 | -0.04306 | 0.00320 | 22484 | -13.46228 | 0.000E+00 | *** |
| Hours6.75 - Hours8.0 | -0.04285 | 0.00320 | 22484 | -13.39516 | 0.000E+00 | *** |

**Supplementary Table S6:** Statistical results from linear mixed-effects model (LMM) for Figure S3C

| **Figure S3C** |  |  |  |  |  |  |
| --- | --- | --- | --- | --- | --- | --- |
| **Contrast** | **Estimate** | **SE** | **df** | **t.ratio** | **p.value** | **Significance** |
| (Hours-1.0) - Hours0.0 | 0.05377 | 0.00208 | 16368 | 25.84432 | 2.550E-08 | *** |
| (Hours-1.0) - Hours0.25 | 0.09102 | 0.00208 | 16368 | 43.75050 | 2.550E-08 | *** |
| (Hours-1.0) - Hours0.5 | 0.09267 | 0.00208 | 16368 | 44.54317 | 2.550E-08 | *** |
| (Hours-1.0) - Hours0.75 | 0.11062 | 0.00208 | 16368 | 53.17237 | 2.550E-08 | *** |
| (Hours-1.0) - Hours1.0 | 0.12322 | 0.00208 | 16368 | 59.23150 | 2.550E-08 | *** |
| (Hours-1.0) - Hours1.25 | 0.12138 | 0.00208 | 16368 | 58.34341 | 2.550E-08 | *** |
| (Hours-1.0) - Hours1.5 | 0.13128 | 0.00208 | 16368 | 63.10535 | 2.550E-08 | *** |
| (Hours-1.0) - Hours1.75 | 0.13652 | 0.00208 | 16368 | 65.62180 | 2.550E-08 | *** |
| (Hours-1.0) - Hours2.0 | 0.13951 | 0.00208 | 16368 | 67.06066 | 2.550E-08 | *** |
| (Hours-1.0) - Hours2.5 | 0.14839 | 0.00208 | 16368 | 71.32668 | 2.550E-08 | *** |
| (Hours-1.0) - Hours2.75 | 0.14767 | 0.00208 | 16368 | 70.98395 | 2.550E-08 | *** |
| (Hours-1.0) - Hours3.0 | 0.15323 | 0.00208 | 16368 | 73.65420 | 2.550E-08 | *** |
| (Hours-1.0) - Hours3.25 | 0.15918 | 0.00208 | 16368 | 76.51644 | 2.550E-08 | *** |
| (Hours-1.0) - Hours3.5 | 0.16459 | 0.00208 | 16368 | 79.11765 | 2.550E-08 | *** |
| (Hours-1.0) - Hours3.75 | 0.17597 | 0.00208 | 16368 | 84.58685 | 2.550E-08 | *** |
| (Hours-1.0) - Hours4.0 | 0.17706 | 0.00208 | 16368 | 85.10917 | 2.550E-08 | *** |
| Hours0.0 - Hours0.25 | 0.03725 | 0.00208 | 16368 | 17.90618 | 2.550E-08 | *** |
| Hours0.0 - Hours0.5 | 0.03890 | 0.00208 | 16368 | 18.69885 | 2.550E-08 | *** |
| Hours0.0 - Hours0.75 | 0.05685 | 0.00208 | 16368 | 27.32805 | 2.550E-08 | *** |
| Hours0.0 - Hours1.0 | 0.06946 | 0.00208 | 16368 | 33.38717 | 2.550E-08 | *** |
| Hours0.0 - Hours1.25 | 0.06761 | 0.00208 | 16368 | 32.49909 | 2.550E-08 | *** |
| Hours0.0 - Hours1.5 | 0.07752 | 0.00208 | 16368 | 37.26102 | 2.550E-08 | *** |
| Hours0.0 - Hours1.75 | 0.08275 | 0.00208 | 16368 | 39.77747 | 2.550E-08 | *** |
| Hours0.0 - Hours2.0 | 0.08575 | 0.00208 | 16368 | 41.21634 | 2.550E-08 | *** |
| Hours0.0 - Hours2.5 | 0.09462 | 0.00208 | 16368 | 45.48235 | 2.550E-08 | *** |
| Hours0.0 - Hours2.75 | 0.09391 | 0.00208 | 16368 | 45.13962 | 2.550E-08 | *** |
| Hours0.0 - Hours3.0 | 0.09946 | 0.00208 | 16368 | 47.80988 | 2.550E-08 | *** |
| Hours0.0 - Hours3.25 | 0.10542 | 0.00208 | 16368 | 50.67211 | 2.550E-08 | *** |
| Hours0.0 - Hours3.5 | 0.11083 | 0.00208 | 16368 | 53.27333 | 2.550E-08 | *** |
| Hours0.0 - Hours3.75 | 0.12221 | 0.00208 | 16368 | 58.74253 | 2.550E-08 | *** |
| Hours0.0 - Hours4.0 | 0.12329 | 0.00208 | 16368 | 59.26485 | 2.550E-08 | *** |
| Hours0.25 - Hours0.5 | 0.00165 | 0.00208 | 16368 | 0.79267 | 1.000E+00 | ns |
| Hours0.25 - Hours0.75 | 0.01960 | 0.00208 | 16368 | 9.42187 | 2.550E-08 | *** |
| Hours0.25 - Hours1.0 | 0.03221 | 0.00208 | 16368 | 15.48100 | 2.550E-08 | *** |
| Hours0.25 - Hours1.25 | 0.03036 | 0.00208 | 16368 | 14.59291 | 2.550E-08 | *** |
| Hours0.25 - Hours1.5 | 0.04027 | 0.00208 | 16368 | 19.35484 | 2.550E-08 | *** |
| Hours0.25 - Hours1.75 | 0.04550 | 0.00208 | 16368 | 21.87129 | 2.550E-08 | *** |
| Hours0.25 - Hours2.0 | 0.04849 | 0.00208 | 16368 | 23.31016 | 2.550E-08 | *** |
| Hours0.25 - Hours2.5 | 0.05737 | 0.00208 | 16368 | 27.57618 | 2.550E-08 | *** |
| Hours0.25 - Hours2.75 | 0.05666 | 0.00208 | 16368 | 27.23344 | 2.550E-08 | *** |
| Hours0.25 - Hours3.0 | 0.06221 | 0.00208 | 16368 | 29.90370 | 2.550E-08 | *** |
| Hours0.25 - Hours3.25 | 0.06817 | 0.00208 | 16368 | 32.76594 | 2.550E-08 | *** |
| Hours0.25 - Hours3.5 | 0.07358 | 0.00208 | 16368 | 35.36715 | 2.550E-08 | *** |
| Hours0.25 - Hours3.75 | 0.08495 | 0.00208 | 16368 | 40.83635 | 2.550E-08 | *** |
| Hours0.25 - Hours4.0 | 0.08604 | 0.00208 | 16368 | 41.35867 | 2.550E-08 | *** |
| Hours0.5 - Hours0.75 | 0.01795 | 0.00208 | 16368 | 8.62920 | 2.550E-08 | *** |
| Hours0.5 - Hours1.0 | 0.03056 | 0.00208 | 16368 | 14.68832 | 2.550E-08 | *** |
| Hours0.5 - Hours1.25 | 0.02871 | 0.00208 | 16368 | 13.80024 | 2.550E-08 | *** |
| Hours0.5 - Hours1.5 | 0.03862 | 0.00208 | 16368 | 18.56217 | 2.550E-08 | *** |
| Hours0.5 - Hours1.75 | 0.04385 | 0.00208 | 16368 | 21.07862 | 2.550E-08 | *** |
| Hours0.5 - Hours2.0 | 0.04684 | 0.00208 | 16368 | 22.51749 | 2.550E-08 | *** |
| Hours0.5 - Hours2.5 | 0.05572 | 0.00208 | 16368 | 26.78350 | 2.550E-08 | *** |
| Hours0.5 - Hours2.75 | 0.05501 | 0.00208 | 16368 | 26.44077 | 2.550E-08 | *** |
| Hours0.5 - Hours3.0 | 0.06056 | 0.00208 | 16368 | 29.11103 | 2.550E-08 | *** |
| Hours0.5 - Hours3.25 | 0.06652 | 0.00208 | 16368 | 31.97326 | 2.550E-08 | *** |
| Hours0.5 - Hours3.5 | 0.07193 | 0.00208 | 16368 | 34.57448 | 2.550E-08 | *** |
| Hours0.5 - Hours3.75 | 0.08331 | 0.00208 | 16368 | 40.04368 | 2.550E-08 | *** |
| Hours0.5 - Hours4.0 | 0.08439 | 0.00208 | 16368 | 40.56599 | 2.550E-08 | *** |
| Hours0.75 - Hours1.0 | 0.01261 | 0.00208 | 16368 | 6.05913 | 2.147E-07 | *** |
| Hours0.75 - Hours1.25 | 0.01076 | 0.00208 | 16368 | 5.17104 | 3.115E-05 | *** |
| Hours0.75 - Hours1.5 | 0.02066 | 0.00208 | 16368 | 9.93298 | 2.550E-08 | *** |
| Hours0.75 - Hours1.75 | 0.02590 | 0.00208 | 16368 | 12.44943 | 2.550E-08 | *** |
| Hours0.75 - Hours2.0 | 0.02889 | 0.00208 | 16368 | 13.88829 | 2.550E-08 | *** |
| Hours0.75 - Hours2.5 | 0.03777 | 0.00208 | 16368 | 18.15431 | 2.550E-08 | *** |
| Hours0.75 - Hours2.75 | 0.03705 | 0.00208 | 16368 | 17.81158 | 2.550E-08 | *** |
| Hours0.75 - Hours3.0 | 0.04261 | 0.00208 | 16368 | 20.48183 | 2.550E-08 | *** |
| Hours0.75 - Hours3.25 | 0.04856 | 0.00208 | 16368 | 23.34407 | 2.550E-08 | *** |
| Hours0.75 - Hours3.5 | 0.05398 | 0.00208 | 16368 | 25.94528 | 2.550E-08 | *** |
| Hours0.75 - Hours3.75 | 0.06535 | 0.00208 | 16368 | 31.41448 | 2.550E-08 | *** |
| Hours0.75 - Hours4.0 | 0.06644 | 0.00208 | 16368 | 31.93680 | 2.550E-08 | *** |
| Hours1.0 - Hours1.25 | -0.00185 | 0.00208 | 16368 | -0.88809 | 1.000E+00 | ns |
| Hours1.0 - Hours1.5 | 0.00806 | 0.00208 | 16368 | 3.87385 | 1.169E-02 | * |
| Hours1.0 - Hours1.75 | 0.01329 | 0.00208 | 16368 | 6.39030 | 4.857E-08 | *** |
| Hours1.0 - Hours2.0 | 0.01629 | 0.00208 | 16368 | 7.82917 | 2.550E-08 | *** |
| Hours1.0 - Hours2.5 | 0.02516 | 0.00208 | 16368 | 12.09518 | 2.550E-08 | *** |
| Hours1.0 - Hours2.75 | 0.02445 | 0.00208 | 16368 | 11.75245 | 2.550E-08 | *** |
| Hours1.0 - Hours3.0 | 0.03000 | 0.00208 | 16368 | 14.42270 | 2.550E-08 | *** |
| Hours1.0 - Hours3.25 | 0.03596 | 0.00208 | 16368 | 17.28494 | 2.550E-08 | *** |
| Hours1.0 - Hours3.5 | 0.04137 | 0.00208 | 16368 | 19.88615 | 2.550E-08 | *** |
| Hours1.0 - Hours3.75 | 0.05275 | 0.00208 | 16368 | 25.35536 | 2.550E-08 | *** |
| Hours1.0 - Hours4.0 | 0.05384 | 0.00208 | 16368 | 25.87767 | 2.550E-08 | *** |
| Hours1.25 - Hours1.5 | 0.00991 | 0.00208 | 16368 | 4.76193 | 2.482E-04 | *** |
| Hours1.25 - Hours1.75 | 0.01514 | 0.00208 | 16368 | 7.27838 | 2.555E-08 | *** |
| Hours1.25 - Hours2.0 | 0.01814 | 0.00208 | 16368 | 8.71725 | 2.550E-08 | *** |
| Hours1.25 - Hours2.5 | 0.02701 | 0.00208 | 16368 | 12.98327 | 2.550E-08 | *** |
| Hours1.25 - Hours2.75 | 0.02630 | 0.00208 | 16368 | 12.64053 | 2.550E-08 | *** |
| Hours1.25 - Hours3.0 | 0.03185 | 0.00208 | 16368 | 15.31079 | 2.550E-08 | *** |
| Hours1.25 - Hours3.25 | 0.03781 | 0.00208 | 16368 | 18.17303 | 2.550E-08 | *** |
| Hours1.25 - Hours3.5 | 0.04322 | 0.00208 | 16368 | 20.77424 | 2.550E-08 | *** |
| Hours1.25 - Hours3.75 | 0.05460 | 0.00208 | 16368 | 26.24344 | 2.550E-08 | *** |
| Hours1.25 - Hours4.0 | 0.05568 | 0.00208 | 16368 | 26.76576 | 2.550E-08 | *** |
| Hours1.5 - Hours1.75 | 0.00524 | 0.00208 | 16368 | 2.51645 | 4.890E-01 | ns |
| Hours1.5 - Hours2.0 | 0.00823 | 0.00208 | 16368 | 3.95532 | 8.546E-03 | ** |
| Hours1.5 - Hours2.5 | 0.01710 | 0.00208 | 16368 | 8.22133 | 2.550E-08 | *** |
| Hours1.5 - Hours2.75 | 0.01639 | 0.00208 | 16368 | 7.87860 | 2.550E-08 | *** |
| Hours1.5 - Hours3.0 | 0.02195 | 0.00208 | 16368 | 10.54886 | 2.550E-08 | *** |
| Hours1.5 - Hours3.25 | 0.02790 | 0.00208 | 16368 | 13.41109 | 2.550E-08 | *** |
| Hours1.5 - Hours3.5 | 0.03331 | 0.00208 | 16368 | 16.01231 | 2.550E-08 | *** |
| Hours1.5 - Hours3.75 | 0.04469 | 0.00208 | 16368 | 21.48151 | 2.550E-08 | *** |
| Hours1.5 - Hours4.0 | 0.04578 | 0.00208 | 16368 | 22.00382 | 2.550E-08 | *** |
| Hours1.75 - Hours2.0 | 0.00299 | 0.00208 | 16368 | 1.43887 | 9.918E-01 | ns |
| Hours1.75 - Hours2.5 | 0.01187 | 0.00208 | 16368 | 5.70488 | 1.619E-06 | *** |
| Hours1.75 - Hours2.75 | 0.01116 | 0.00208 | 16368 | 5.36215 | 1.113E-05 | *** |
| Hours1.75 - Hours3.0 | 0.01671 | 0.00208 | 16368 | 8.03241 | 2.550E-08 | *** |
| Hours1.75 - Hours3.25 | 0.02266 | 0.00208 | 16368 | 10.89464 | 2.550E-08 | *** |
| Hours1.75 - Hours3.5 | 0.02808 | 0.00208 | 16368 | 13.49586 | 2.550E-08 | *** |
| Hours1.75 - Hours3.75 | 0.03945 | 0.00208 | 16368 | 18.96506 | 2.550E-08 | *** |
| Hours1.75 - Hours4.0 | 0.04054 | 0.00208 | 16368 | 19.48737 | 2.550E-08 | *** |
| Hours2.0 - Hours2.5 | 0.00887 | 0.00208 | 16368 | 4.26601 | 2.397E-03 | ** |
| Hours2.0 - Hours2.75 | 0.00816 | 0.00208 | 16368 | 3.92328 | 9.676E-03 | ** |
| Hours2.0 - Hours3.0 | 0.01372 | 0.00208 | 16368 | 6.59354 | 3.151E-08 | *** |
| Hours2.0 - Hours3.25 | 0.01967 | 0.00208 | 16368 | 9.45577 | 2.550E-08 | *** |
| Hours2.0 - Hours3.5 | 0.02508 | 0.00208 | 16368 | 12.05699 | 2.550E-08 | *** |
| Hours2.0 - Hours3.75 | 0.03646 | 0.00208 | 16368 | 17.52619 | 2.550E-08 | *** |
| Hours2.0 - Hours4.0 | 0.03755 | 0.00208 | 16368 | 18.04851 | 2.550E-08 | *** |
| Hours2.5 - Hours2.75 | -0.00071 | 0.00208 | 16368 | -0.34273 | 1.000E+00 | ns |
| Hours2.5 - Hours3.0 | 0.00484 | 0.00208 | 16368 | 2.32752 | 6.341E-01 | ns |
| Hours2.5 - Hours3.25 | 0.01080 | 0.00208 | 16368 | 5.18976 | 2.821E-05 | *** |
| Hours2.5 - Hours3.5 | 0.01621 | 0.00208 | 16368 | 7.79097 | 2.550E-08 | *** |
| Hours2.5 - Hours3.75 | 0.02759 | 0.00208 | 16368 | 13.26018 | 2.550E-08 | *** |
| Hours2.5 - Hours4.0 | 0.02867 | 0.00208 | 16368 | 13.78249 | 2.550E-08 | *** |
| Hours2.75 - Hours3.0 | 0.00556 | 0.00208 | 16368 | 2.67026 | 3.760E-01 | ns |
| Hours2.75 - Hours3.25 | 0.01151 | 0.00208 | 16368 | 5.53249 | 4.322E-06 | *** |
| Hours2.75 - Hours3.5 | 0.01692 | 0.00208 | 16368 | 8.13371 | 2.550E-08 | *** |
| Hours2.75 - Hours3.75 | 0.02830 | 0.00208 | 16368 | 13.60291 | 2.550E-08 | *** |
| Hours2.75 - Hours4.0 | 0.02939 | 0.00208 | 16368 | 14.12522 | 2.550E-08 | *** |
| Hours3.0 - Hours3.25 | 0.00595 | 0.00208 | 16368 | 2.86224 | 2.543E-01 | ns |
| Hours3.0 - Hours3.5 | 0.01137 | 0.00208 | 16368 | 5.46345 | 6.362E-06 | *** |
| Hours3.0 - Hours3.75 | 0.02274 | 0.00208 | 16368 | 10.93265 | 2.550E-08 | *** |
| Hours3.0 - Hours4.0 | 0.02383 | 0.00208 | 16368 | 11.45497 | 2.550E-08 | *** |
| Hours3.25 - Hours3.5 | 0.00541 | 0.00208 | 16368 | 2.60121 | 4.255E-01 | ns |
| Hours3.25 - Hours3.75 | 0.01679 | 0.00208 | 16368 | 8.07041 | 2.550E-08 | *** |
| Hours3.25 - Hours4.0 | 0.01788 | 0.00208 | 16368 | 8.59273 | 2.550E-08 | *** |
| Hours3.5 - Hours3.75 | 0.01138 | 0.00208 | 16368 | 5.46920 | 6.162E-06 | *** |
| Hours3.5 - Hours4.0 | 0.01246 | 0.00208 | 16368 | 5.99152 | 3.125E-07 | *** |
| Hours3.75 - Hours4.0 | 0.00109 | 0.00208 | 16368 | 0.52232 | 1.000E+00 | ns |
